# Anisotropic Micro/Nanotopography Regulating Mitochondrial Dynamics in Cardiomyocytes

**DOI:** 10.34133/research.0891

**Published:** 2025-09-16

**Authors:** Yan Liu, Bingcheng Yi, Liangliang Yang, Yanyan Yang, Tianxiang Li, Xiaolu Li, Jae Youl Cho, Dengshen Zhang, Qihui Zhou, Tao Yu

**Affiliations:** ^1^ Institute for Translational Medicine, The Affiliated Hospital of Qingdao University, Qingdao 266021, People’s Republic of China.; ^2^ Department of Integrative Biotechnology, Sungkyunkwan University, Suwon 16419, Republic of Korea.; ^3^ Qingdao Key Laboratory of Materials for Tissue Repair and Rehabilitation, Shandong Engineering Research Center for Tissue Rehabilitation Materials and Devices, School of Rehabilitation Sciences and Engineering, University of Health and Rehabilitation Sciences, Qingdao 266113, People’s Republic of China.; ^4^ School of Pharmaceutical Sciences, Wenzhou Medical University, Wenzhou 325035, People’s Republic of China.; ^5^ Department of Immunology, School of Basic Medicine, Qingdao University, Qingdao 266021, Shandong, People’s Republic of China.; ^6^ Department of Cardiac Ultrasound, the Affiliated Hospital of Qingdao University, Qingdao 266000, Shandong, People’s Republic of China.; ^7^ Department of Cardiovascular Surgery, Affiliated Hospital of Zunyi Medical University, Zunyi 563000, Guizhou, People’s Republic of China.

## Abstract

**Introduction:** Topographical cues of biomaterial scaffolds directly guide cell behaviors by determining integrin ligation and subsequent mechanotransducive pathways, but their influence on organelle (e.g., mitochondrion) behaviors remains unclear. **Objectives:** Considering the high sensitivity of mitochondria in cardiomyocytes to topographical signals, this study focused on investigating the impact of oriented micro/nano-wrinkled surfaces with varying wavelengths (0.5 to 25.0 μm) and amplitudes (0.05 to 4.30 μm) on the mitochondrial functions of rat embryonic myocardial cell line H9c2. **Methods and Results:** The results uncover a nonlinear response of cardiomyocyte behavior and mitochondrial homeostasis to these surface features. Notably, surfaces with a 3-μm wavelength and 0.7-μm amplitude (W3) promoted substantial cell elongation and orientation, whereas surfaces with a 0.5-μm wavelength and 0.05-μm amplitude (W0.5) triggered pronounced mitochondrial division. Remarkably, W0.5 topography facilitated mitochondrial division via cytoskeletal remodeling, involving vinculin and tubulin, which disrupted mitochondrial energy metabolism, enhanced reactive oxygen species (ROS)-mediated oxidative stress, and perturbed mitochondrial homeostasis by stimulating the adenosine 5′-monophosphate-activated protein kinase (AMPK) pathway. The transcriptomic analysis identifies the pivotal involvement of the p53, FoxO, mTOR, HIF-1, and AMPK signaling pathways in regulating mitochondrial dynamics in myocardial cells induced by W0.5, confirming the essential role of the polyadenylation signal (AATAAA) in modulating transcript splicing processes. **Conclusion:** Overall, this study offers important insights into the regulatory mechanisms by which aligned micro/nano topographical stimuli impact mitochondrial responses in cardiomyocytes, which hold potential for the development of novel biomaterial-focused approaches for diagnosing and treating cardiovascular diseases.

## Introduction

The substrate microenvironment is essential in regulating cell behaviors during biomaterial-guided in situ tissue regeneration [1,2]. Focal adhesion-mediated sensing of physical and functional cues within the matrix allows cells to transduce these signals into intracellular signaling pathways, ultimately influencing cell growth, differentiation, and apoptosis [3,4]. Thus, cell–matrix interactions are crucial in tissue remodeling and repairing. In the area of tissue engineering utilizing biomaterials, the development of biomimetic scaffolds possessing suitable physicochemical characteristics, such as topographical [5–7], biochemical [8,9], and mechanical [10,11] signals, has surfaced as a hopeful approach for the regeneration of functional tissues [12]. Surface topography, which refers to specific morphological features of scaffolds in direct contact with cells, is a prominent physical cue that affects cellular responses [13,14]. Recent research studies have shown that the characteristics of surface topography can influence cellular behavior by influencing the cellular cytoskeleton to exert forces on the nucleus or by indirectly altering mechanosensitive pathways through integrin receptor-related biochemical signaling on the cell membrane [2,15]. Despite significant progress in understanding the regulation of cell behavior by surface topography, the mechanisms underlying the impact of micro/nanotopographic cues of biomaterial scaffolds on organelles, such as mitochondria, have been largely overlooked. Given that mitochondria primarily function to generate adenosine triphosphate (ATP) energy and store calcium for cell signaling activities, which in turn control cell growth and death [16,17], a deeper understanding of how substrate topographical features influence mitochondria can provide valuable insights into the progress of scaffolds made from biomaterials in the field of regenerative medicine.

Mitochondrion-associated cellular energy metabolism is closely linked to ATP production through oxidative phosphorylation [18]. The ATP generated powers various cellular activities, including biomacromolecule formation (such as nucleotides, lipids, and iron–sulfur clusters), cell growth, cell death, chronic inflammation, and stem cell differentiation [17,19]. Recently, numerous research indicated that mitochondrial damage is associated with most heart diseases [20–22]. For example, mitochondrial disorders can lead to reduced energy production, increased oxidative stress, and interference with apoptosis signaling pathways [23,24], resulting in abnormal apoptosis processes in cardiomyocytes [25]. Additionally, damage to mitochondrial DNA or failure of the mitochondrial respiratory chain can cause the release of free electrons in mitochondria, leading to high levels of reactive oxygen species (ROS), which can induce mitochondrial toxicity [26]. Furthermore, impaired mitochondrial function can activate the inflammatory response of the immune system and contribute to tissue fibrosis, thereby causing varying degrees of damage to heart tissues [27]. Moreover, due to the abundance of mitochondria, myocardial cells exhibit high sensitivity to topographical signals on substrates [28]. Therefore, the construction of an effective mitochondrial quality control (QC) system is essential for the biomaterial-mediated therapy of myocardial injury [29].

Recently, the arrangement of cardiomyocytes on wrinkled surfaces has been demonstrated to enhance the growth and differentiation of heart tissue for repair purposes [30]. However, it remains unclear whether the topographical structure provides a mechanical cue to modulate the mitochondria-associated bioenergetics of myocardial cells. To investigate this, we designed wrinkled polydimethylsiloxane (PDMS) surfaces with varying sizes, ranging from nanometers to micrometers, to explore the effects on cardiomyocyte morphology and intracellular environment, and reveal the related regulatory mechanisms. This study aims to model the process in which cardiomyocytes experience external pressure during their functioning. The fabrication of wrinkled PDMS surfaces with anisotropic micro/nanotopography was achieved by using an elastomer base (prepolymer) and cross-linker (Sylgard 184) in a weight ratio of 10:1, as previously described [31]. Following the seeding of H9c2 cells, molecular biotechnologies, including immunofluorescent staining and Western blot (WB) analysis, were employed to investigate the mitochondrial homeostasis and oxidative stress response in the myocardial cells. Additionally, transcriptome analysis was conducted to uncover the regulatory mechanisms underlying the behaviors of H9c2 cells in response to the micro/nano-wrinkled topography.

## Results and Discussion

### Micro/nano-wrinkled topographical surface significantly affects cardiomyocyte cell morphology

**Fig. 1.**
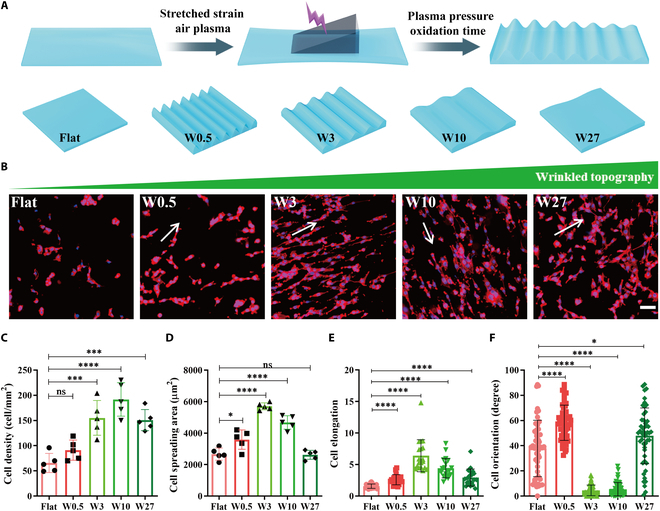
Micro/nano-wrinkled topographical surface significantly affects cardiomyocyte cell morphology. (A) Schematic of PDMS substrates. (B) Cell morphology of H9c2 cells on wrinkled PDMS substrates at 24 h. Red and blue fluorescence represent F-actin and nuclei, respectively. Scale bar, 20 μm. (C to F) Quantitative analysis (*n* > 50) of cell density, cell spreading area, cell elongation, and cell orientation. *P < 0.05, ***P < 0.005, ****P < 0.001.

Due to the relaxation of the stretched polymer after the release of strain, PDMS substrates were successfully induced to form micro/nano-wrinkled topographical surfaces through mechanical stretching and plasma treatment [7]. The topographic characteristics of PDMS can be controlled by adjusting the degree of stretched strain and the conditions of the air plasma, such as plasma pressure and oxidation time (Fig. 1A). The micro/nanotopographical surfaces of PDMS are shown in atomic force microscopy (AFM) images in Fig. S1. Typically, when cells adhere to substrates, they can sense the surface topography of the substrate through focal adhesions, which are binding proteins between cell transmembrane molecules and the material. This allows the cells to transduce the sensed physical signals from the microenvironment into intracellular signaling pathways, thereby influencing cytoskeleton remodeling and cell spreading, a phenomenon referred to as “contact guidance” [2]. As illustrated in Fig. 1B, a notable difference in cell morphology was observed between the wrinkled topography and smooth surface. Specifically, quantitative analysis of cell density revealed a significant increase in cell number on the W10 surface compared to the Flat PDMS (Fig. 1C), attributed to the increased specific surface area of the wrinkled topography, which provides more adhesion sites for cell attachment compared to the smooth surface [20]. However, the most considerable cell spreading area was observed in the W3 group (Fig. 1D). Increasing the wrinkle size from Flat, W0.5, W3, W10, and W27 resulted in the highest cell elongation along the direction of the wrinkled topography in the W3 group, accompanied by the lowest cell orientation (Fig. 1E and F). This suggests that W3 provides optimal contact guidance for cell spreading. Moreover, it is speculated that topography-mediated cell spreading influences cell responses, as accumulating evidence has demonstrated that the rearrangement of the cytoskeleton induced by topographical cues can exert mechanical stimuli on organelles and the nucleus, ultimately influencing gene expression changes [32–34].

### The wrinkled topography of W0.5 facilitates mitochondrial division by modulating cytoskeletal remodeling

**Fig. 2.**
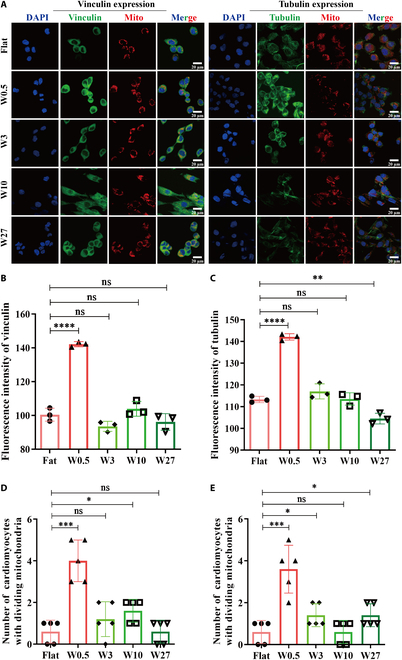
The wrinkled topography of W0.5 facilitates mitochondrial division by modulating cytoskeletal remodeling. (A) Immunofluorescent staining of vinculin, tubulin, and mitochondria in H9c2 cells. (B) Quantified fluorescence intensity of vinculin. (C) Quantified fluorescence intensity of tubulin. (D and E) Quantified number of fragmented mitochondrial in H9c2 cells from vinculin- and tubulin-based images, respectively. The detected mitochondria include punctate in the division state and linear in the fusion state. *P < 0.05, **P < 0.01, ****P < 0.001.

**Fig. 3.**
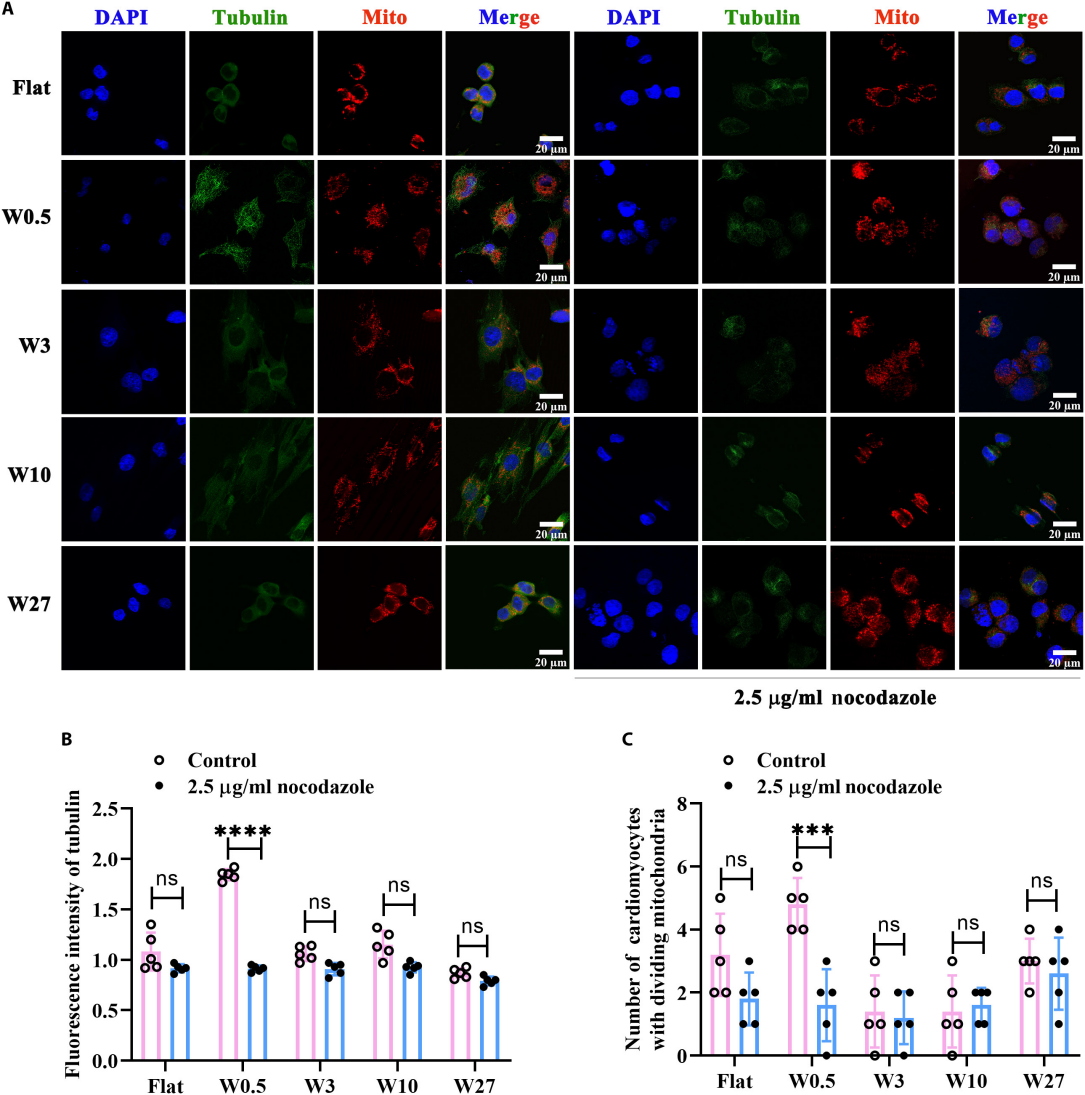
The wrinkled topography of W0.5 facilitates mitochondrial division by modulating cytoskeletal remodeling. (A) Immunofluorescent staining of tubulin and mitochondria in H9c2 cells treated with 2.5 μg/ml nocodazole and their control. (B) Quantified fluorescence intensity of tubulin. (C) Quantified number of fragmented mitochondrial in H9c2 cells from tubulin-based images. The detected mitochondria include punctate in the division state and linear in the fusion state. *P < 0.05, **P < 0.01, ****P < 0.001.

Given the close association between cytoskeletal remodeling and mitochondrial transport, morphological plasticity, and function [35,36], it can be inferred that topographical surfaces that regulate cell morphology have the potential to impact the morphology and function of mitochondria in H9c2 cells. Consequently, the expression of cytoskeletal components (i.e., vinculin and tubulin), along with fragmented mitochondria, was assessed in H9c2 cells [37]. As anticipated, while all groups demonstrated noticeable vinculin and tubulin expression on the PDMS substrate (Fig. 2A), the highest expression was observed on the W0.5 surface (Fig. 2B and C). This suggests that W0.5 promotes significant cytoskeletal remodeling in H9c2 cells compared to the other PDMS groups. However, this trend contrasts with the observed cell morphology, where the highest cell alignment was observed on W3. This discrepancy is mainly due to the combined effect of space-controlled contact guidance and focal adhesion-guided cytoskeletal remodeling on topography-induced changes in cell morphology [14]. On W3, contact guidance may have a more dominant influence on directing cell morphology compared to cytoskeletal remodeling. To further investigate the mechanical stimulation of cytoskeleton rearrangement on mitochondria, mitochondria fission in H9c2 cells was examined through immunofluorescent staining of MitoTracker Red. As depicted in Fig. 2A, clear mitochondrial fragmentation was observed on W0.5 (Fig. 2D and E). We performed the functional perturbation experiments in Fig. 3A to C, disrupting the cytoskeleton with nocodazole, observing the expression of tubulin and mitochondrial dynamics. Our observations revealed that treatment with nocodazole significantly suppressed tubulin expression in the presence of W0.5. Additionally, nocodazole markedly mitigated the mitochondrial fission triggered by W0.5, thus confirming the relationship between mitochondrial division and cytoskeletal remodeling. In conclusion, these findings support the notion that the wrinkled topography of W0.5 promotes mitochondrial division by regulating cytoskeletal remodeling.

### The wrinkled topography of W0.5 significantly perturbs mitochondrial homeostasis

**Fig. 4.**
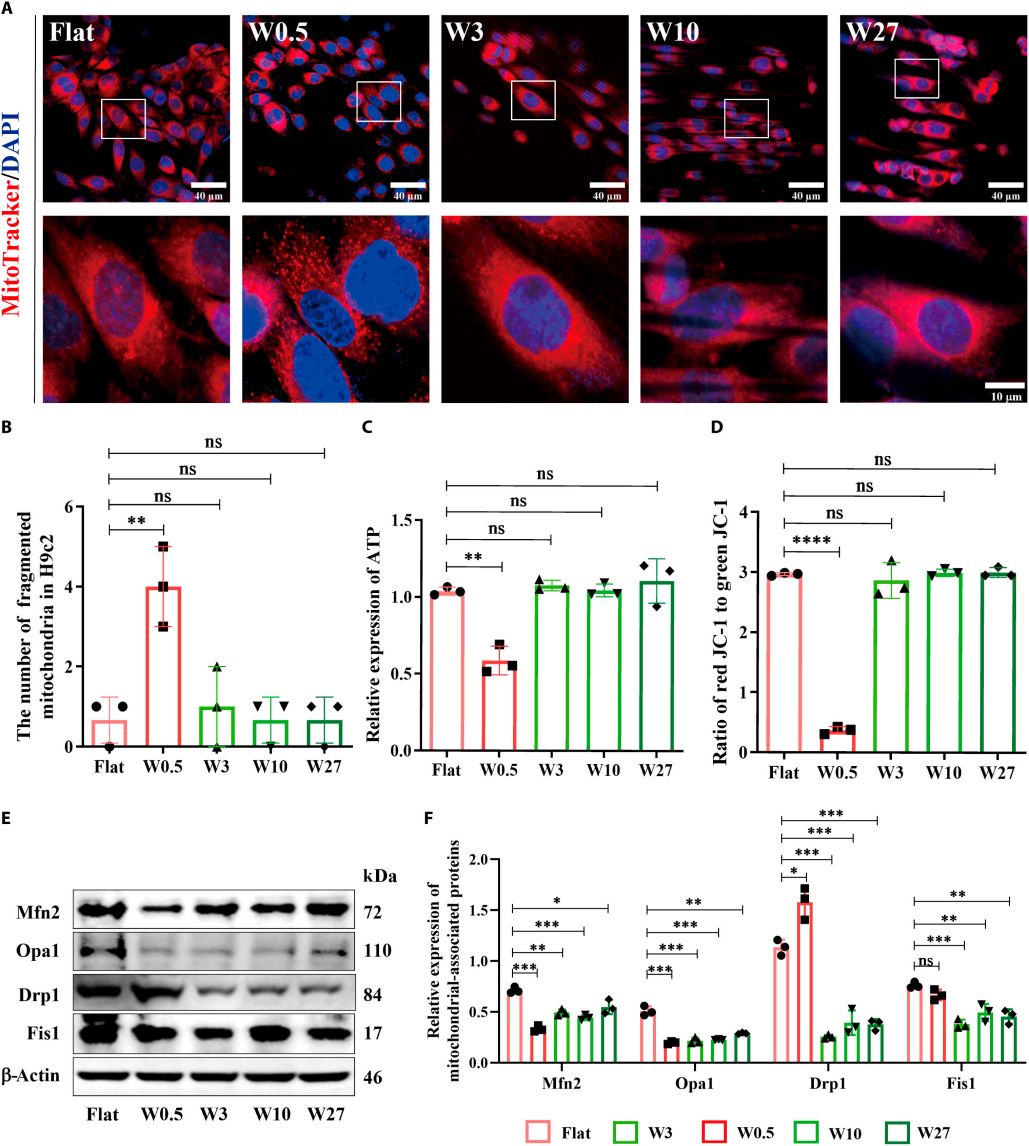
The wrinkled topography of W0.5 significantly perturbs mitochondrial homeostasis. (A) Immunofluorescent staining of mitochondria in H9c2 cells using MitoTracker Red. (B) Quantified number of fragmented mitochondrial in H9c2 cells from (A). (C) ATP production in H9c2. (D) Detection of JC-1 level for mitochondrial membrane potential. (E) Expression of mitochondrial homeostasis-related proteins (Mfn2, Opa1, Drp1, and Fis1) in H9c2 cells. (F) Quantitative analysis of protein expression (*n* = 3). *P < 0.05, **P < 0.01, ***P < 0.005, ****P < 0.001.

Mitochondria are significant dynamism organelles that maintain mitochondrial network homeostasis through continuous fusion and division [38]. This procedure is essential in preserving energy metabolism and contractile function in cardiomyocytes. Dysregulation of mitochondrial dynamics can lead to mitochondrial rupture and contribute to the pathological development of cardiovascular diseases, such as dilated cardiomyopathy, ischemia–reperfusion injury, and heart failure [39–41]. In this study, we observed an initial increase followed by a decrease in mitochondrial fragmentation in myocardial cells with increasing wrinkle sizes from Flat, W0.5, W3, W10, and W27 (Fig. 4A), with the most pronounced mitochondrial fragmentation occurring on W0.5 (Fig. 4B). Meanwhile, during mitochondrial fusion and division, Drp1, a cytosolic protein, collaborates with mitochondrial outer membrane proteins (such as Fis1 and mitochondrial fission factors) to facilitate the division process [42,43]. On the other hand, the process of mitochondrial fusion is regulated by Mfn2 located in the outer mitochondrial membrane and Opa1 found in the inner mitochondrial membrane [44]. To analyze mitochondrial homeostasis on a molecular level, the expression of mitochondrial fusion/division-related proteins in H9c2 cells was assessed by WB. As shown in Fig. 4E and F, compared to a smooth surface, the wrinkled topography of W0.5 significantly suppressed the expression of Mfn2 and Opa1, while not significantly affecting the mitochondrial fission proteins (i.e., Drp1 and Fis1). In addition, the primary role of mitochondria is to generate ATP for cellular metabolism [45]. Any impairment in mitochondrial dynamics can lead to ATP biogenesis defects and disrupt mitochondrial energy metabolism [46]. Accordingly, the W0.5 wrinkled topography significantly inhibited ATP production (Fig. 4C). Furthermore, mitochondria store electrochemical potential energy in their inner membrane during energy generation, and this energy generates the mitochondrial membrane potential when there is an asymmetric concentration of protons or ions across the inner membrane [47]. In other words, increased energy production is associated with higher mitochondrial membrane potential [48]. This was further confirmed by the lower expression of JC-1 (a fluorescent indicator for measuring mitochondrial membrane potential) on W0.5 compared to other groups (Fig. 4D). This finding confirms that the wrinkled topography of W0.5 is prone to disrupting the dynamic equilibrium between mitochondrial fusion and division.

**Fig. 5.**
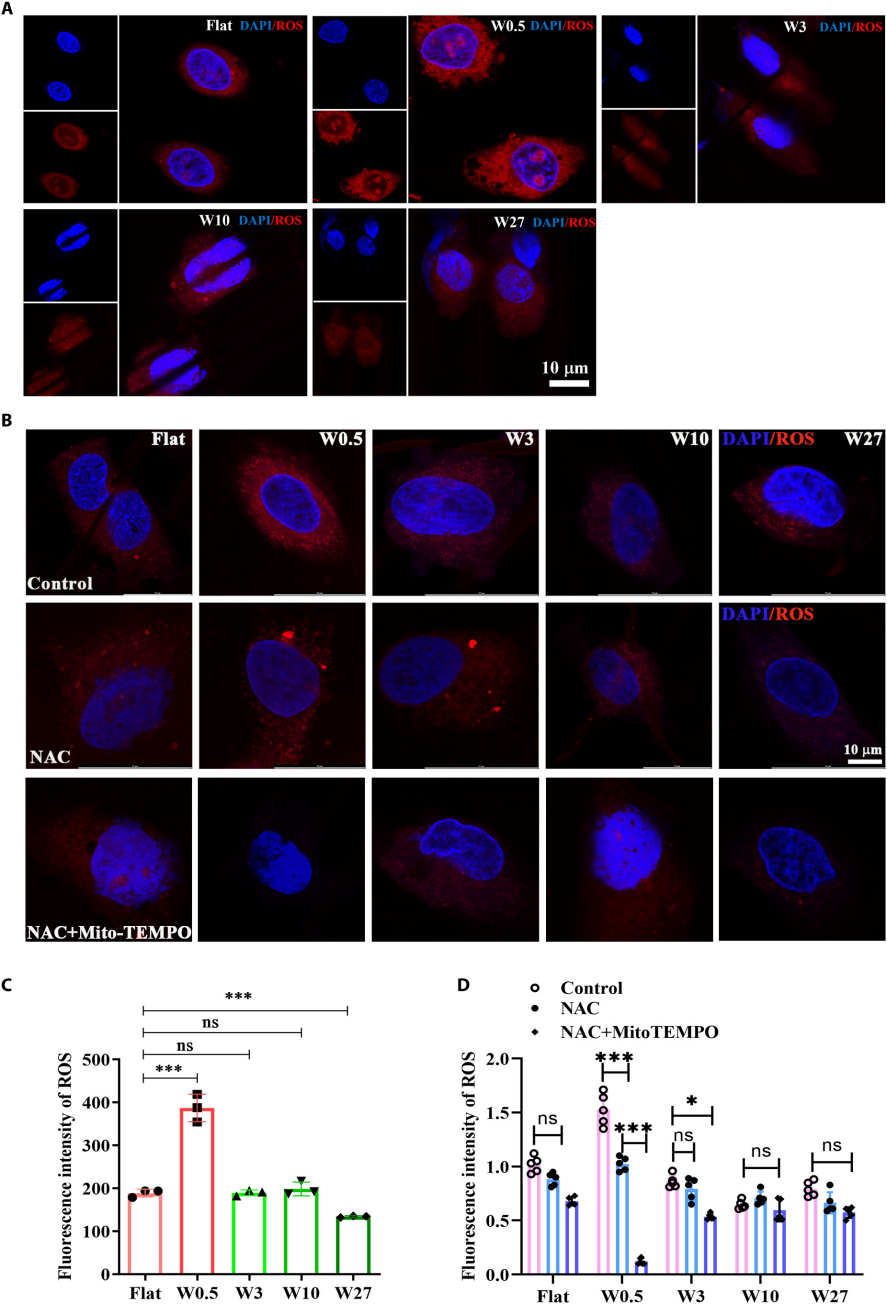
The wrinkled topography of W0.5 significantly perturbs ROS production. (A) Immunofluorescent staining of ROS in H9c2 cells. (B) Immunofluorescent staining of ROS in H9c2 cells treated with NAC and control. (C) Quantified ROS fluorescence in H9c2 cells from (A). *N* = 3. (D) Quantified ROS fluorescence in H9c2 cells from (B). *N* = 5. ***P < 0.005.

Additionally, when mitochondria are stimulated by external stimuli, mitochondrial ROS (mROS) produced during oxidative phosphorylation as a consequence of bioenergetic metabolism can induce oxidative stress, cellular senescence, and severe diseases [49–51]. Consistent with mitochondrial fragmentation, higher levels of ROS were induced in the W0.5 group compared to the other groups (Fig. 5A and C). Notably, normal cells produce minimal ROS. Although our study emphasizes how mitochondrial fission promotes ROS production, the mutual regulatory role of ROS in mitochondrial dynamics is worth noting [52]. ROS are established activators of fission pathways, primarily through the posttranslational modification and activation of DRP1 (e.g., via phosphorylation at serine-616) [53]. This ROS–DRP1 axis promotes mitochondrial fragmentation, creating a potential feedback loop wherein fission-derived ROS further amplifies fission [54]. Our findings that increased fission correlates with oxidative stress suggest a unidirectional relationship. Future studies should investigate whether targeting the ROS–DRP1 interaction could modulate mitochondrial dysfunction observed in H9c2 cells under varying wrinkle sizes, particularly W0.5. Neglecting this reciprocal relationship may oversimplify the complex interplay between redox signaling and mitochondrial morphology. To ensure that W0.5 can indeed cause mitochondrial fission. We used the mitochondrial-targeted antioxidant mitoTEMPO (1 μM) [55] and the general antioxidant *N*-acetylcysteine (NAC) (1 mM) [56], treated H9C2 grown on different wrinkles of PDMS. The results showed that NAC and mitoTEMPO could effectively reduce the production of ROS in the W0.5 group (Fig. 5B and D).

Taken together, we use the above indicators (fusion/fission index, ATP levels, membrane potential JC-1, and ROS production) to evaluate mitochondrial homeostasis. These findings demonstrated that the W0.5 wrinkled topography significantly disrupts mitochondrial energy metabolism, increases mROS-induced oxidative stress, and consequently disturbs mitochondrial homeostasis [57].

### The wrinkled topography of W0.5 disrupts mitochondrial homeostasis by activating the AMPK pathway

**Fig. 6.**
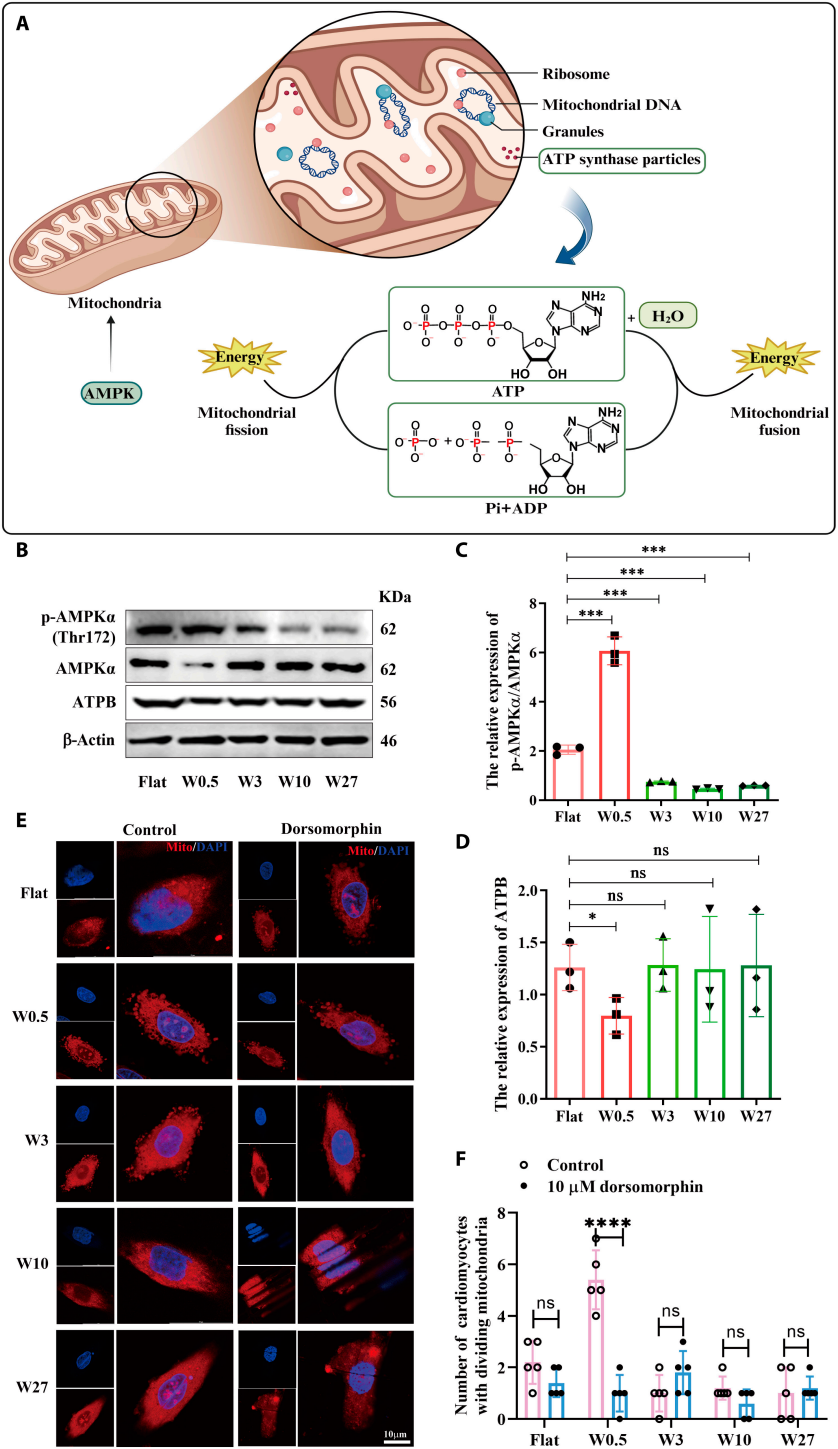
The wrinkled topography of W0.5 disrupts mitochondrial homeostasis by activating the AMPK pathway. (A) ATP consumption and role of AMPK during mitochondrial fission and fusion. (B) Expression of AMPK pathway-related proteins (AMPKα, p-AMPKα, and ATPB). (C) Quantitative analysis of AMPKα/p-AMPKα ratio. (D) Quantitative analysis of ATPB. (E) Administered the AMPK inhibitor dorsomorphin (10 μM) to H9c2 cells, confocal observation of mitochondrial status. (F) Quantified number of fragmented mitochondria in H9c2 cells. *N* ≥ 3. ***P < 0.005, ****P < 0.001.

Accumulating evidence demonstrates that cells develop complex biological systems, including the activation of adenosine 5′-monophosphate (AMP)-activated protein kinase (AMPK) complexes, to cope with energy stress [58,59]. For instance, AMPK phosphorylates specific enzymes and growth control nodes to enhance ATP generation and reduce ATP consumption under low-energy conditions [60,61]. Therefore, the W0.5-induced decrease in ATP levels in H9c2 cells is likely to induce comparable energy stress and trigger the fragmented mitochondrial phenotype by affecting AMPK activation (Fig. 6A). Because AMPKα is closely related to the ATP energy supply reduction, AMPK signal pathway is activated, and the phosphorylated AMPKα (p-AMPKα)/total AMPKα are always keeping the balance [62]. This phenomenon accurately confirmed that AMPKα and p-AMPKα play a regulating role in the mitochondrial energy supply process. Consistently, the increased p-AMPKα/AMPKα ratio and decreased expression of ATPB (ATP synthase) observed in the W0.5 group (Fig. 6B to D) further confirm the disruption of mitochondrial homeostasis by regulating the AMPK pathway. To further elucidate the role of W0.5 in disrupting mitochondrial homeostasis through AMPK activation, we administered the AMPK inhibitor dorsomorphin (10 μM) [63] to H9c2 cells and monitored the resulting mitochondrial dynamics. Our results demonstrated that dorsomorphin significantly mitigated the mitochondrial fission induced by W0.5, while other cellular morphological features remained unaffected (Fig. 6E and F). These findings align with our previous conclusions. This indicates that the W0.5 wrinkled topography primarily inhibits mitochondrial fusion, leading to the disruption of mitochondrial homeostasis. Conversely, increasing the size of the wrinkled topography (such as W3, W10, and W27) tended to simultaneously suppress the fusion and division of mitochondria, thereby maintaining mitochondrial dynamics balance.

### The potential signaling pathway and involved cardiovascular diseases regulated by W0.5

**Fig. 7.**
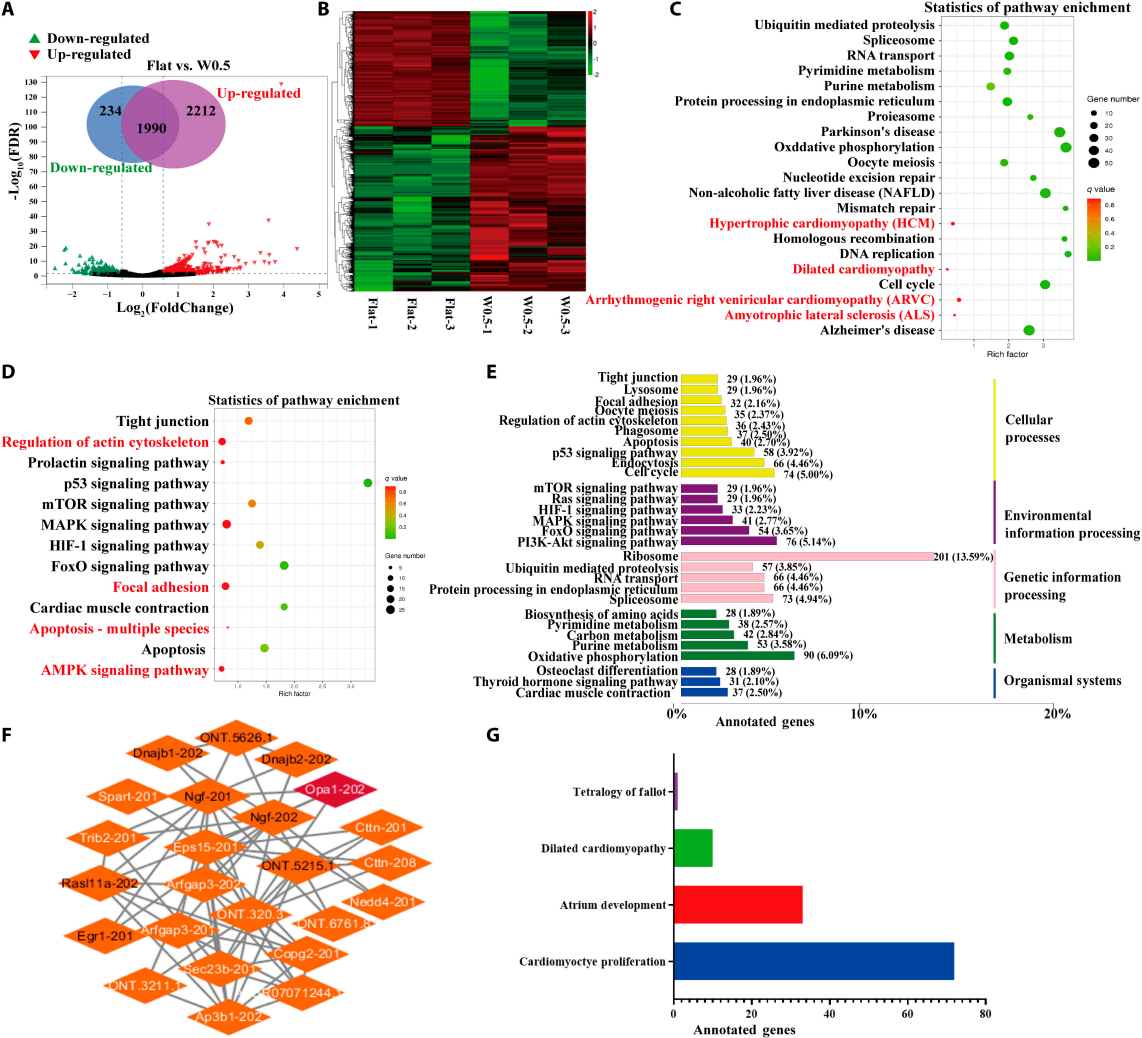
The potential signaling pathway and involved cardiovascular diseases regulated by W0.5. (A) The volcano plots were performed to provide a quick visualization method of transcripts and genes, displaying the expression alternation between the W0.5-treated group and the Flat group. (B) The hierarchical cluster indicated the difference between the W0.5-treated group and the Flat group. (C and D) Statistics of pathway enrichment in DETs, including up-regulation and down-regulation. (E) The GO annotation results of the DETs were classified according to the pathway types in GO. (F) Using the STRING database to analyze the protein–protein interaction network of Opa1. (G) KEGG analysis of the DEGs. *N* ≥ 3, *P* ≤ 0.05.

To further compare the gene expression profiles at transcriptional levels, cells in W0.5 and Flat groups were sequenced by transcript sequencing following the experiment workflow (Fig. S2, *n* = 3). Based on the criteria of fold change greater than 1.5 and a *P* value of less than 0.05, a total of 2,446 transcripts differentially expressed genes (DEGs) were identified as significantly altered, in which 2,212 transcripts were up-regulated and 234 were down-regulated (Fig. 7A). The hierarchical cluster confirmed the significant difference in expression pattern of the altered transcripts and genes between the W0.5 group and the control group (Fig. 7B and Fig. S3A to D). Next, we performed database function annotation for differentially expressed transcripts (DETs) and DEGs. The statistics of the annotated transcripts showed that cellular process and binding activity account for most systems in DETs and DEGs (Fig. S3E). The Gene Ontology (GO) annotation results of the DETs were classified according to the pathway types in GO, which displays the top 20 pathways with the smallest significant *Q* value. We observed that ribosome process, spliceosome, p53 signaling pathway, cell cycle, and oxidative phosphorylation are the most predominant signaling in DETs (Fig. S3F). Particularly, the transcripts related to cell cycle, apoptosis, and cardiac muscle contraction exhibit the strongest dysregulation after W0.5 treatment in cardiomyocytes. The transcriptional sequencing results implied that W0.5 higher probability changes the morphology and basic function of cardiomyocytes.

To illustrate whether W0.5 biomaterials participate in the putative role in the pathogenesis of cardiovascular diseases and its mechanism, we analyzed the potential signaling pathways. Hypertrophic cardiomyopathy, arrhythmogenic right ventricular cardiomyopathy, and dilated cardiomyopathy are close to the regulation of W0.5 PDMS (Fig. 7C). Then, the top 20 underlying pathways related to biomaterial functions were analyzed. The signals involved in the actin cytoskeleton, tight junction, focal adhesion, and cardiac muscle contraction are significantly regulated (Fig. 7D and E), while the apoptosis and ATP metabolism pathways, including p53 signaling, FoxO signaling, mTOR (mammalian target of rapamycin) signaling, HIF-1 (hypoxia-inducible factor 1) signaling, and AMPK signaling, were the main pathways mediated by W0.5 PDMS. Transcriptome analysis suggests concurrent activation of stress-responsive pathways (AMPK, p53) and metabolism-related pathways (mTOR, HIF-1, FoxO). We propose these form a “mechano-metabolic signaling network” with AMPK as a central hub linking cytoskeletal remodeling to mitochondrial dynamics. Moreover, as OPA1 was proved to be significantly regulated by W0.5 PDMS in our previous study, we then performed the protein–protein interaction network of OPA1 by using the STRING database, including the direct (physical) interactions and indirect (functional) associations (Fig. 7F). We found that Opa1 mainly binds nerve growth factor (NGF) and other newly identified genes (ONT.5215.1) to regulate biological functions in cardiomyocytes. Subsequently, we used the Kyoto Encyclopedia of Genes and Genomes (KEGG) analysis to observe the role of DEGs in regulating the disease, and the results showed that DEGs are mainly involved in cardiomyocyte proliferation and atrium development (Fig. 7G). Besides, since fusion genes can lead to sequence abnormalities, gene expression disorders, and protein dysfunction, which can lead to or promote the occurrence of various diseases, we used MinION sequencing to identify fusion transcripts. The results showed that there is no obvious fusion among these target genes, including Opa1, Drp1, Fis1, Mfn1, Mfn2, AMPK, and ATPB. These results showed that W0.5 participated in the mitochondrial homeostasis of cardiomyocytes.

### Gene structure and alternative splicing analysis

**Fig. 8.**
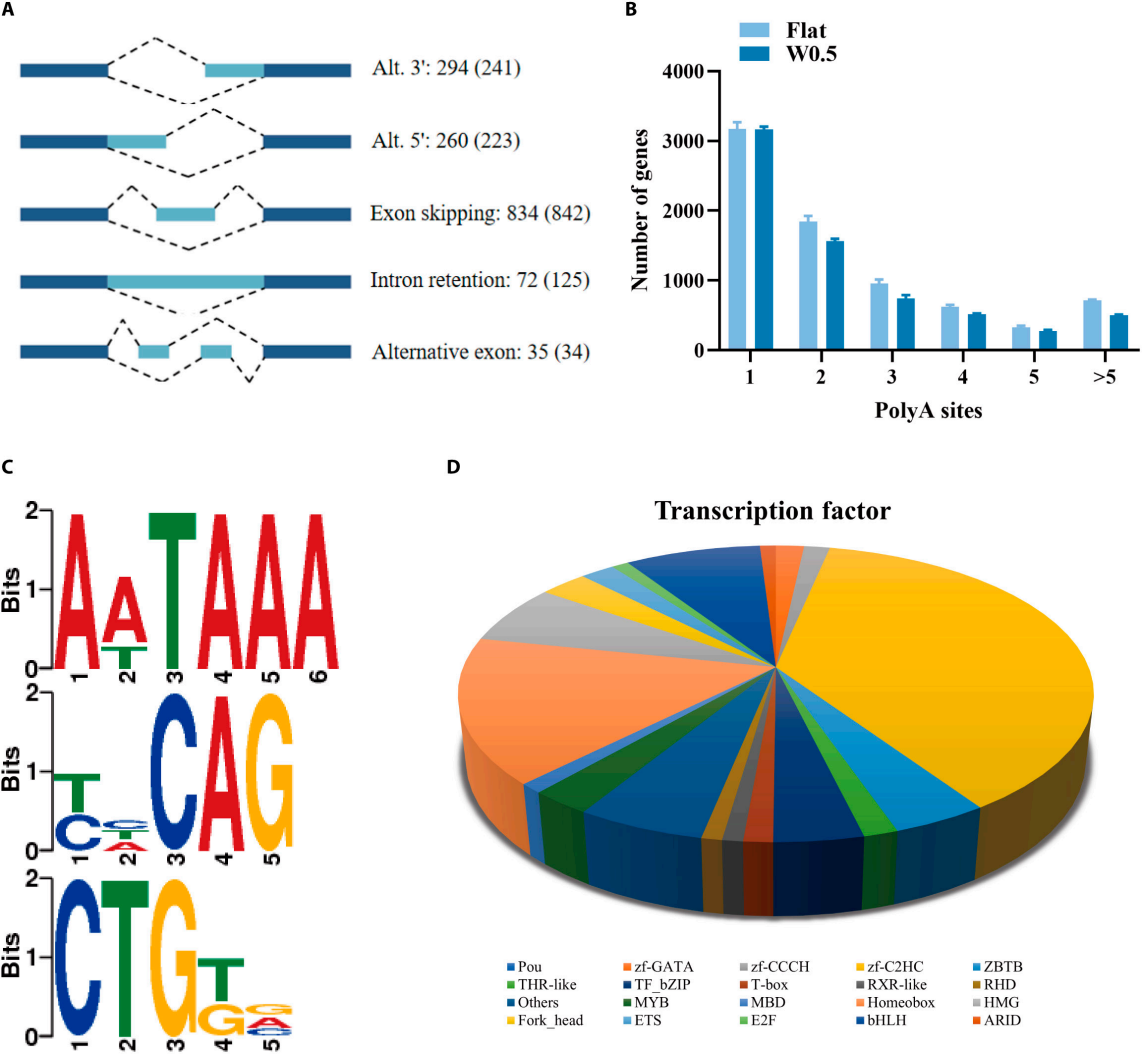
Gene structure and alternative splicing analysis. (A) Alternative splicing of DETs by using Astalavista software. (B and C) Analyze FLNC to identify APA using TAPIS pipeline. (D) Analyze the binding of RNA polymerase to DNA templates using animal TFDB 2.0.

Due to the limitation of the software or data, the annotation of the selected reference genome is often not accurate enough. Therefore, it is necessary to optimize the original annotated gene structure. Meanwhile, we further explore the different mechanisms of W0.5-treated groups causing the decrease in total transcript numbers. As a method capable of accurately identifying the structure of transcripts, full-length sequencing was used. Gffcomp is used to align transcripts obtained by full-length sequencing to known transcripts of the genome, so novel genes and transcripts can be obtained to supplement genome annotation. Firstly, we analyzed the alternative splicing of DETs by using Astalavista software and statistics of the 5 alternative splicing events of transcripts. We found that exon skipping events account for the majority of splices (Fig. S4), and there are 241 alternative 3′ splice site and 223 alternative 5′ splice site combinations in W0.5-treated groups, which are significantly lower than controls, whereas the 125 intron retention events revealed obviously higher than controls (Fig. 8A). The different alternative splicing would result in different translations of proteins and eventually bring diversity to biological traits in W0.5 and Flat groups.

In prokaryotes, the alternative polyadenylation (APA) of precursor mRNA may play a role in enhancing transcriptome diversity, influencing genome coding, and regulating gene expression. Therefore, we conduct a further analysis of full-length nonchimeric (FLNC) sequences to identify instances of APA using the TAPIS pipeline (transcriptome analysis pipeline from isoform sequencing). As shown in Fig. 8B, transcripts from over 4,200 genes contain 2 or more polyadenylation sites, suggesting that APA is a common phenomenon in cardiomyocytes. Interestingly, the W0.5 treatment strongly reduced to 3,500 genes, which might explain the decrease of total number of transcripts. This analysis identified a significantly overrepresented polyadenylation signal (AATAAA) (Fig. 8C). In addition, the TCCAG and CTGTG motifs were also found around 50 nucleotides upstream of the poly(A) site (Fig. 8C). Then, we analyzed transcription factors as they have the ability to control the attachment of RNA polymerase to DNA templates, thus influencing gene transcription through the use of animal TFDB 2.0 (animal transcription factor database). A total of 1,603 transcription factors were predicted, and the prediction results are shown in Fig. 8D. Zf-C2H2, a family of zinc finger E-box binding (ZEB) homeobox that could regulate cell cycle, cell death, and aging and embryo development through repressing transcription, occupied the most abundance of differential transcription factors. This evidence explains the reason for the decrease in total transcript numbers in W0.5-treated groups from different mechanisms (Fig. 8).

In addition, since lncRNA (long noncoding RNA) does not code for proteins, the transcripts were screened to determine whether it had coding potential; thus, we can determine whether the transcript is lncRNA or not. Meanwhile, long noncoding RNAs were also reported to be closely related to cardiovascular diseases. Therefore, we analyzed the differentially expressed lncRNAs regulated by W0.5 in this study via analysis of CPC, CNCI, CPAT, and PFAM protein domains. A total of 325 lncRNA transcripts were predicted by all 4 methods (Fig. S5A). We made an lncRNA classification map based on the location of lncRNA within the annotation details of the reference genome (Fig. S5B).

Based on our in vitro experiments and transcriptome sequencing results, we emphasize that anisotropic micro/nanotopography acts as a physical cue that directly influences cellular mechanotransduction via focal adhesion–integrin interactions and cytoskeletal remodeling. Our work bridges a critical gap by demonstrating how these cues propagate to organelle-level responses (mitochondria), an underexplored area in biomaterial design. Simultaneously, we correlated our results with cardiac pathology and noted mitochondrial fragmentation on the W0.5 wrinkle, which may serve as a distinctive marker for myocardial diseases such as ischemic cardiomyopathy and heart failure [64]. The disruption of the fusion/fission balance caused by W0.5 exemplifies the in vivo stress response, underscoring its utility in modeling disease mechanisms. Our data indicate that cytoskeletal remodeling, evidenced by increased vinculin and tubulin levels, acts as a catalyst for mitochondrial fission. However, it is important to recognize that excessive ROS production might independently drive fission through Drp1 activation. Additionally, the heightened ROS levels observed on W0.5 surfaces may not be exclusively due to mitochondrial impairment; they could also directly induce fission via redox-sensitive phosphorylation of Drp1 [54]. Employing ROS scavengers like NAC helped elucidate the underlying causative mechanisms. We explicitly state that H9c2 cells (rat-derived) may not fully recapitulate human cardiomyocyte responses. Transcriptomic pathways (e.g., p53 and FoxO) require validation in primary human cells or cardiac tissues. Also, we propose that W3/W10 topographies (promoting cell alignment without fission) are optimal for cardiac patches, while W0.5 serves as a platform to study pathological mitochondrial fragmentation.

## Conclusion

In the present study, we used micro/nanotopography of PDMS to interact with H9c2 and observed that the W0.5 wrinkled substrate disrupts mitochondrial homeostasis, leading to abnormal fission of H9c2 mitochondria. At the same time, an oxidative stress reaction occurred in H9c2, and a large number of ROS were produced. To more intuitively understand the effect of fold substrate on the mitochondrial division of H9c2, we detected the expressions of Mfn2, Opa1, Drp1, and Fis1 and found that W0.5 significantly increased the expression of mitochondrial fission protein Drp1. It also causes the activation of the AMPK pathway. It can also affect the cytoskeletal structure, increasing the expression of tubulin and vinculin. To enhance the reliability of our findings, we conducted a transcriptome sequencing analysis, which, through KEGG analysis, confirmed that W0.5 plays a role in the regulatory mechanism of cardiomyocytes. In this study, topological structures of different wrinkle surfaces were used to simulate the growth environment of cardiomyocytes and participate in the regulation and repair of cardiomyocytes. The results showed that W3 and W10 were more suitable for the growth of cardiomyocytes, while W0.5 had an obvious destructive effect.

Our conclusions are primarily derived from in vitro models, which may not fully recapitulate the complexity of intact myocardium. Although mechanistically informative, direct clinical implications remain speculative without in vivo validation. Future investigations into employing rat models will be critical to establish pathophysiological relevance. It is worth noting that W3/W10 topographies, which promote cell alignment without disrupting mitochondria (Figs. 1E and 4B), have potential application value in optimizing the design of cardiac patches for myocardial repair.

## Materials and Methods

### Fabrication of micro/nano-wrinkled PDMS

The precursor solution of PDMS was prepared with an elastomer base (prepolymer, Sigma-Aldrich) and Sylgard 184 (cross-linker, Dow Corning) in a weight ratio of 10:1, following the provided instructions. The blend was thoroughly mixed using a spatula and subsequently degassed in a vacuum for 15 min. Subsequently, the mixture was deposited onto a clean 12 × 12 cm polystyrene petri dish. The sample was cured at 70 °C overnight to obtain a Flat PDMS substrate. To create wrinkled PDMS, the Flat PDMS substrate obtained was uniaxially stretched to a strain of 10% to 30% of its original length using a custom-made stretching apparatus, as described in our previous study [65]. The elongated substrate was subsequently subjected to oxidation through air plasma (Plasma Activate Flecto 10 USB, at maximum intensity) under varying pressure conditions (25 mtorr and 14 torr) and oxidation times (20 to 1,800 s), following the methodology outlined in our previous study [31]. Finally, the strain applied to the PDMS was released, resulting in the generation of micro/nano-wrinkled PDMS with various morphological features (i.e., W0.5A0.05, W3A0.7, W10A3.5, and W27A4.3), which were assigned as W0.5, W3, W10, and W27, respectively. In this notation, “W” denotes the wavelength of the wrinkled topography, while “A” represents the amplitude. The specific values of surface topography for each sample are as follows: W0.5 (wavelength: 0.5 μm, amplitude: 0.05 μm), W3 (wavelength: 3 μm, amplitude: 0.7 μm), W10 (wavelength: 10 μm, amplitude: 3.5 μm), and W27 (wavelength: 27 μm, amplitude: 4.3 μm).

#### AFM of micro/nano-wrinkled PDMS

AFM imaging was conducted utilizing a commercial NanoScope V Dimension 3100 microscope (Veeco, USA) operating in tapping mode under ambient conditions. The wavelength and amplitude of the wrinkles observed in these images were quantitatively analyzed using NanoScope Analysis software. The modulus of the samples was determined using a BioScope Catalyst AFM instrument (Bruker, Billerica, MA, USA) integrated with Nano Scope Analysis software. All measurements were executed in quantum-mechanical nano-mapping mode with high amplitude, employing Bruker SCANASYST-AIR cantilevers fabricated from silicon nitride with silicon tips.

### Effect of micro/nano-wrinkled topography on cell morphology

#### Cell culture and seeding

H9c2 cells were sourced from the American Type Culture Collection (USA) and were grown in Dulbecco’s modified Eagle’s medium (Meilunbio, China) enriched with 10% fetal bovine serum (ExCell Bio, China) and 1% penicillin–streptomycin (Invitrogen, USA). The cells were incubated at 37 °C in a 5% CO_2_ atmosphere [66]. For cell seeding, the PDMS substrates were sterilized and placed in 24-well culture plates. Subsequently, H9c2 cells at 90% confluence were harvested and seeded onto the substrates as per the experimental requirements. The drug-treated cells are as follows: nocodazole (Oncodazole, MedChemExpress, MCE, China, R17934; 2.5 μg/ml), NAC (MedChemExpress, MCE, China, HY-B0215; 1 mM), MitoTEMPO (MedChemExpress, MCE, China, HY-112879, 1 μM), and dorsomorphin (compound C, MedChemExpress, MCE, China, BML-275; 10 μM).

#### Observation of cell morphology

H9c2 cells were cultured on PDMS substrates at a density of 2 × 10^5^ cells per well in a 24-well plate. After 24 h of incubation, the cells were rinsed 3 times using phosphate-buffered saline (PBS; Solarbio, China) and then fixed at room temperature with 4% paraformaldehyde (Solarbio, China) for 20 min. Following this, a solution of 0.5% Triton X-100 (Solarbio, China) was introduced for 10 min to increase cell membrane permeability. The cells were then stained with 200 nM tetramethyl rhodamine isothiocyanate–phalloidin (Sigma, USA) for 30 min and 10 μg/ml 4′,6-diamidino-2-phenylindole (DAPI; Solarbio, China) for 10 min. Finally, the cell morphology was visualized using fluorescence microscopy (Nikon A1 MP, Japan). For quantitative analysis, parameters including cell density (cell number per square millimeter), cell spreading area, cell elongation (the ratio of the major axis to the minor axis of cells), and cell orientation (the angle between the cell elongation direction and the substrate wrinkle direction) were directly calculated using ImageJ software, following previous studies [10,31].

#### Analysis of cytoskeleton and mitochondria remodeling

The topological structure of the substrate influences cell morphology by regulating focal adhesions that connect the cell cytoskeleton to the substrate [2]. Since the cytoskeleton directly impacts mitochondrial behavior [35], immunofluorescence staining of vinculin and α-tubulin was performed in combination with mitochondrial labeling using MitoTracker Red CMXRos (Invitrogen M7512, USA), following the provided instructions. Briefly, H9c2 cells were cultured on the wrinkled PDMS substrates at a density of 2 × 10^5^ cells per well in a 24-well plate for 24 h. After washing with PBS 3 times, the cells were co-incubated with 125 nM MitoTracker Red CMXRos for 20 min at 37 °C, followed by fixation with 4% paraformaldehyde for 30 min. Subsequently, a 0.5% solution of Triton X-100 (Solarbio, Beijing, China) was applied to enhance the permeability of the cell membrane for 20 min at room temperature. To prevent nonspecific binding, the cells were treated with 3% bovine serum albumin (BSA) in PBS for 30 min. Next, the cells were incubated with primary antibodies (α-tubulin: Cell Signaling Technology, USA, 1:2,000; anti-vinculin antibody: Abcam, USA, 1:100) for 2 h at room temperature, followed by incubation with secondary fluorescent antibodies [fluorescein isothiocyanate (FITC)-conjugated AffinPure Goat Anti-Mouse IgG (H+L), Jackson ImmunoResearch, UK, 1:50; FITC-conjugated AffinPure Goat Anti-Rabbit IgG (H+L), Jackson ImmunoResearch, UK, 1:50] for 1 h at room temperature. The expression levels of tubulin and vinculin, along with the mitochondrial condition in H9c2 cells, were examined using a confocal laser scanning microscope (Leica, TCS SPE, Germany). The analysis of cardiomyocytes with dividing mitochondria was performed using ImageJ software (Java 1.8.0, USA). Fragmented mitochondria were defined as punctuate structures, while fused mitochondria were tubular. This method aligns with established protocols [67].

### Effect of micro/nano-wrinkled topography on mitochondrial functions

#### ATP expression assay

ATP expression was quantified using an ATP detection kit (Beyotime, China) that utilizes firefly luciferase to catalyze fluorescein production, resulting in fluorescence emission [68]. H9c2 cells were seeded on PDMS substrates at a density of 2 × 10^5^ cells per well in a 24-well plate and incubated for 24 h. After washing the cells 3 times with PBS, the total protein was collected using ATP lysis buffer from the kit, and the protein concentration was determined using a bicinchoninic acid (BCA) Protein Assay Kit (Sigma, USA). The total protein was then aliquoted into black 96-well plates (JingAn Biological, China). Subsequently, 20 μl of the protein sample was mixed with 100 μl of the ATP working solution following the kit’s instructions. The ATP expression was measured using an Enzyme Labeling Instrument (BioTek, USA) to quantify the relative light units. The relative ATP expression levels were analyzed using GraphPad Prism 5 software (GraphPad Software Inc., USA).

#### Detection of mitochondrial membrane potential

Mitochondrial membrane potential was assessed using the JC-1 mitochondrial fluorescent probe. H9c2 cells were seeded on PDMS substrates at a density of 2 × 10^5^ cells per well in a 24-well plate and incubated at 37 °C in a 5% CO_2_ incubator for 24 h. The cells were then stained with JC-1 mitochondrial fluorescent probe (10 μg/ml, Yeasen, China) for 15 min at 37 °C. After washing the cells 3 times with PBS, trypsin was used to detach the cells and form cell suspensions. Subsequently, 100 μl of the cell suspension was added to black 96-well plates. The levels of mitochondrial membrane potential in normal cells (represented by red fluorescence) and apoptotic/unhealthy cells (represented by green fluorescence) were measured using a microplate reader. Finally, the ratio of red fluorescence to green fluorescence was calculated to analyze mitochondrial homeostasis.

#### Observation of ROS production

ROS production in H9c2 cells was assessed using the MitoSOX Red Mitochondrial Superoxide Indicator (Yeasen, China). H9c2 cells were seeded on PDMS substrates at a density of 2 × 10^5^ cells per well in a 24-well plate and incubated at 37 °C in a 5% CO_2_ incubator for 24 h. The cells were then stained with 5 μM MitoSOX Red Mitochondrial Superoxide Indicator and 10 μg/ml DAPI solution. Subsequently, the H9c2 cells were washed 3 times with PBS, and the expression of ROS was visualized using a confocal laser scanning microscope. The quantitative analysis of ROS expression, represented by fluorescence intensity per cell, was performed using ImageJ software based on the fluorescence microscopy images.

### Effect of micro/nano-wrinkled topography on mitochondrial homeostasis

#### Determination of mitochondrial division

To investigate the association of mitochondrial dynamics with dynamin-related protein 1 (Drp1), mitochondrial fission 1 (Fis1), mitochondrial fusion 2 (Mfn2), and optic atrophy 1 (Opa1) [69], the protein expressions of these factors were evaluated using WB analysis. H9c2 cells were seeded on PDMS substrates at a density of 2 × 10^5^ cells per well in a 24-well plate and incubated at 37 °C in a 5% CO_2_ incubator for 48 h. Total proteins were extracted from the H9c2 cells using immunoprecipitation assay with radioimmunoprecipitation assay (RIPA) lysis buffer (Solarbio, China) containing phenylmethanesulfonyl fluoride (Solarbio, China) and protease inhibitor cocktail (Abmole China Branch, China). The concentration of protein was measured using a BCA Protein Assay Kit. Following this, 30 μg of protein was isolated by subjecting them to 12.5% sodium dodecyl sulfate–polyacrylamide gel electrophoresis (SDS-PAGE). The proteins were then transferred onto a polyvinylidene difluoride (PVDF) membrane (Millipore, USA) and blocked with 5% BSA (Invitrogen, USA) at room temperature for 1 h on a shaking platform. The PVDF membrane was then incubated with primary antibodies followed by secondary antibodies, each for 1 h. Protein bands were visualized using enhanced chemiluminescence (ECL) luminophores (EpiZyme, SQ201, China), and the protein expressions were quantitatively analyzed using ImageJ software. The details of the primary antibodies used are as follows: rabbit anti-FIS1 polyclonal antibody (1:2,000, Absin, China), rabbit anti-DRP1 monoclonal antibody (1:1,000, Cell Signaling Technology, USA), rabbit anti-OPA1 polyclonal antibody (1:2,000, Abcam, USA), and rabbit anti-mitofusin-2 monoclonal antibody (1:1,000, Cell Signaling Technology, USA). Rabbit anti-β-actin monoclonal antibody (1:1,000, Cell Signaling Technology, USA) was used as a reference protein. The secondary antibodies used were goat anti-rabbit IgG (H+L) (1:5,000, Absin, China).

#### Verification of AMPK pathway involved in mitochondrial energy metabolism

To investigate the involvement of AMPK pathway in mitochondrial fragmentation and analyze mitochondrial energy metabolism, a WB assay was also performed following the protocol outlined in previously described method. The related first antibodies were as follows: rabbit anti-phospho-AMPKα (Thr^172^) antibody (1:1,000, Cell Signaling Technology, USA), rabbit anti-AMPKα antibody (1:1,000, Cell Signaling Technology, USA), and rabbit anti-ATPB antibody (1:10,000, Abcam, USA).

### Transcriptome analysis of H9c2 cells regulated by micro/nano-wrinkled topography

#### Experiment workflow of transcriptome analysis

The experiment workflow followed the standard protocol provided by Oxford Nanopore Technologies, which involved sample QC, library construction, library QC, and library sequencing [70]. The workflow consisted of the following steps: Firstly, total RNA from H9c2 cells treated with PDMS substrates was isolated using TRIzol reagent (Sigma, Darmstadt, Germany) on dry ice. The RNA was then reverse transcribed using SuperScript IV reverse transcriptase. The resulting cDNA was purified and concentrated using AMPure XP beads. In the second step, cDNA libraries were generated from 50 ng of total RNA utilizing the cDNA-PCR Sequencing Kit (SQK-PCS109) and the PCR Barcoding Kit (SQK-PBK004), following a 14-cycle polymerase chain reaction (PCR) amplification as per the manufacturer’s instructions (Oxford Nanopore Technologies Ltd., Oxford, UK). Subsequently, the resulting cDNA libraries were sequenced using the PromethION system (Oxford Nanopore Technologies Ltd., Oxford, UK) by Biomarker Technologies (Beijing, China). Three biological replicates were performed (*n* = 3). To obtain the full-length transcriptome, the analysis process involved 3 steps [71]: full-length sequence recognition, consensus sequence polishing, and consensus sequence level clustering.

#### Long read processing

The raw reads were subjected to filtering, requiring an average read Q score of at least 7 and a minimum length of 500 base pairs. After filtering, reading and mapping to the rRNA database were discarded as they represented ribosomal RNA. FLNC transcripts were identified by detecting primers at both ends of the reads. Clusters of FLNC transcripts were generated by aligning them to the reference genome using minimap2. Within each cluster, consensus isoforms were obtained by applying polishing using pinfish (https://github.com/nanoporetech/pinfish). The consensus sequences were subsequently aligned to the reference genome (assembly AAP 1.0) utilizing minimap2. The mapped reads were then further processed using the cDNA_Cupcake package (https://github.com/Magdoll/cDNA_Cupcake) with a minimum coverage threshold of 85% and a minimum identity threshold of 90%. When collapsing redundant transcripts, differences in the 5′ end were not taken into consideration.

### Statistical analyses

All data used at least 3 independent experiments and were presented as the mean ± SE, which were analyzed by GraphPad Prism 5 software. In the specific analysis process, the Student’s *t* test was used for the significance test between the 2 different groups. A *P* value of <0.05 (*P < 0.05, **P < 0.01, ***P < 0.005, ****P < 0.001) was considered a statistically significant difference.

## Data Availability

Data are available on request from the corresponding author.

## References

[1] Guan Y, Racioppi L, Gerecht S. Engineering biomaterials to tailor the microenvironment for macrophage–endothelium interactions. Nat Rev Mater. 2023;8(10):688–699.

[2] Yi B, Xu Q, Liu W. An overview of substrate stiffness guided cellular response and its applications in tissue regeneration. Bioact Mater. 2022;15:82–102. 35386347 10.1016/j.bioactmat.2021.12.005 PMC8940767

[3] Maihofer J, Madry H, Rey-Rico A, Venkatesan JK, Goebel L, Schmitt G, Speicher-Mentges S, Cai X, Meng W, Zurakowski S, et al. Hydrogel-guided, rAAV-mediated IGF-I overexpression enables long-term cartilage repair and protection against Perifocal osteoarthritis in a large-animal full-thickness chondral defect model at one year in vivo. Adv Mater. 2021;33(16): Article e2008451. 33734514 10.1002/adma.202008451 PMC11468525

[4] Li Y, Xiao Y, Liu C. The horizon of Materiobiology: A perspective on material-guided cell behaviors and tissue engineering. Chem Rev. 2017;117(5):4376–4421. 28221776 10.1021/acs.chemrev.6b00654

[5] Nikkhah M, Edalat F, Manoucheri S, Khademhosseini A. Engineering microscale topographies to control the cell–substrate interface. Biomaterials. 2012;33(21):5230–5246. 22521491 10.1016/j.biomaterials.2012.03.079 PMC3619386

[6] Zhou Q, Castañeda Ocampo O, Guimarães CF, Kühn PT, van Kooten TG, van Rijn P. Screening platform for cell contact guidance based on inorganic biomaterial micro/nanotopographical gradients. ACS Appl Mater Interfaces. 2017;9(37):31433–31445. 28825457 10.1021/acsami.7b08237 PMC5609122

[7] Zhou Q, Wünnemann P, Kühn PT, de Vries J, Helmin M, Böker A, van Kooten TG, van Rijn P. Mechanical properties of aligned Nanotopologies for directing cellular behavior. Adv Mater Interfaces. 2016;3(18):1600275.

[8] Yi B, Zhou B, Song Z, Yu L, Wang W, Liu W. Step-wise CAG@PLys@PDA-Cu2+ modification on micropatterned nanofibers for programmed endothelial healing. Bioact Mater. 2023;25:657–676. 37056258 10.1016/j.bioactmat.2022.07.010 PMC10086768

[9] Yi B, Yu L, Tang H, Wang W, Liu W, Zhang Y. Lysine-doped polydopamine coating enhances antithrombogenicity and endothelialization of an electrospun aligned fibrous vascular graft. Appl Mater Today. 2021;25: Article 101198.

[10] Yi B, Shen Y, Tang H, Wang X, Li B, Zhang Y. Stiffness of aligned fibers regulates the phenotypic expression of vascular smooth muscle cells. ACS Appl Mater Interfaces. 2019;11(7):6867–6880. 30676736 10.1021/acsami.9b00293

[11] Yi B, Shen Y, Tang H, Wang X, Zhang Y. Stiffness of the aligned fibers affects structural and functional integrity of the oriented endothelial cells. Acta Biomater. 2020;108:237–249. 32205213 10.1016/j.actbio.2020.03.022

[12] Ghosh B, Nong J, Wang Z, Urban MW, Heinsinger NM, Trovillion VA, Wright MC, Lepore AC, Zhong Y. A hydrogel engineered to deliver minocycline locally to the injured cervical spinal cord protects respiratory neural circuitry and preserves diaphragm function. Neurobiol Dis. 2019;127:591–604. 31028873 10.1016/j.nbd.2019.04.014 PMC6588451

[13] Karazisis D, Rasmusson L, Petronis S, Palmquist A, Shah FA, Agheli H, Emanuelsson L, Johansson A, Omar O, Thomsen P. The effects of controlled nanotopography, machined topography and their combination on molecular activities, bone formation and biomechanical stability during osseointegration. Acta Biomater. 2021;136:279–290. 34626821 10.1016/j.actbio.2021.10.001

[14] Yi B, Zhou B, Dai W, Lu X, Liu W. Soft nanofiber modified micropatterned substrates enhance native-like endothelium maturation via CXCR4/calcium-mediated actin cytoskeleton assembly. Nano Res. 2023;16(1):792–809.

[15] Fraire JC, Guix M, Hortelao AC, Ruiz-Gonzalez N, Bakenecker AC, Ramezani P, Hinnekens C, Sauvage F, De Smedt SC, Braekmans K, et al. Light-triggered mechanical disruption of extracellular barriers by swarms of enzyme-powered Nanomotors for enhanced delivery. ACS Nano. 2023;17(8):7180–7193. 37058432 10.1021/acsnano.2c09380 PMC10134497

[16] Szabo I, Szewczyk A. Mitochondrial ion channels. Annu Rev Biophys. 2023;52:229–254. 37159294 10.1146/annurev-biophys-092622-094853

[17] Nichtova Z, Fernandez-Sanz C, De La Fuente S, Yuan Y, Hurst S, Lanvermann S, Tsai H-Y, Weaver D, Baggett R, Thompson C, et al. Enhanced mitochondria-SR tethering triggers adaptive cardiac muscle remodeling. Circ Res. 2023;132(11):e171–e187. 37057625 10.1161/CIRCRESAHA.122.321833 PMC10213149

[18] Oliveira AG, Oliveira LD, Cruz MV, Guimarães DSPSF, Lima TI, Santos-Fávero BC, Luchessi AD, Pauletti BA, Leme AP, Bajgelman MC, et al. Interaction between poly-a binding protein PABPC4 and nuclear receptor corepressor NCoR1 modulates a metabolic stress response. J Biol Chem. 2023;299(6):104702. 37059182 10.1016/j.jbc.2023.104702 PMC10203745

[19] Jin H, Liu AD, Holmberg L, Zhao M, Chen S, Yang J, Sun Y, Chen S, Tang C, du J. The role of sulfur dioxide in the regulation of mitochondrion-related cardiomyocyte apoptosis in rats with isopropylarterenol-induced myocardial injury. Int J Mol Sci. 2013;14(5):10465–10482. 23698774 10.3390/ijms140510465 PMC3676849

[20] Piscioneri A, Morelli S, Ritacco T, Giocondo M, Peñaloza R, Drioli E, de Bartolo L. Topographical cues of PLGA membranes modulate the behavior of hMSCs, myoblasts and neuronal cells. Colloids Surf B Biointerfaces. 2023;222: Article 113070. 36495697 10.1016/j.colsurfb.2022.113070

[21] Krestinin R, Baburina Y, Odinokova I, Kruglov A, Sotnikova L, Krestinina O. The effect of Astaxanthin on mitochondrial dynamics in rat heart mitochondria under ISO-induced injury. Antioxidants. 2023;12(6):1247. 37371979 10.3390/antiox12061247 PMC10295417

[22] Li Y, Peng X, Lin R, Wang X, Liu X, Meng F, Ruan Y, Bai R, Tang R, Liu N. Tyrosine kinase inhibitor antitumor therapy and atrial fibrillation: Potential off-target effects on mitochondrial function and cardiac substrate utilization. CVIA J. 2023;8(1):944.

[23] Ijaz MU, Shahzadi S, Hamza A, Azmat R, Anwar H, Afsar T, Shafique H, Bhat MA, Naglah AM, al-Omar MA, et al. Alleviative effects of pinostrobin against cadmium-induced renal toxicity in rats by reducing oxidative stress, apoptosis, inflammation, and mitochondrial dysfunction. Front Nutr. 2023;10:1175008. 37342552 10.3389/fnut.2023.1175008 PMC10278233

[24] Peng H, Yao F, Zhao J, Zhang W, Chen L, Wang X, Yang P, Tang J, Chi Y. Unraveling mitochondria-targeting reactive oxygen species modulation and their implementations in cancer therapy by nanomaterials. Exploration. 2023;3(2):20220115. 37324035 10.1002/EXP.20220115 PMC10191003

[25] Bei Y, Wang H, Liu Y, Su Z, Li X, Zhu Y, Zhang Z, Yin M, Chen C, Li L, et al. Exercise-induced miR-210 promotes cardiomyocyte proliferation and survival and mediates exercise-induced cardiac protection against ischemia/reperfusion injury. Research. 2024;7:0327. 38410280 10.34133/research.0327 PMC10895486

[26] Zhu H, Dai Z, Liu X, Zhou H, Wang Y. Serine/threonine kinase 3 promotes oxidative stress and mitochondrial damage in septic cardiomyopathy through inducing Kelch-like ECH-associated protein 1 phosphorylation and nuclear factor erythroid 2-related factor 2 degradation. Int J Biol Sci. 2023;19(5):1369–1381. 37056939 10.7150/ijbs.80800 PMC10086747

[27] Gonzalez-Armenta JL, Bergstrom J, Lee J, Furdui CM, Nicklas BJ, Molina AJA. Serum factors mediate changes in mitochondrial bioenergetics associated with diet and exercise interventions. Geroscience. 2023;46(1):349–365. 37368157 10.1007/s11357-023-00855-w PMC10828137

[28] Tallawi M, Dippold D, Rai R, ’Atri D D, Roether JA, Schubert DW, Rosellini E, Engel FB, Boccaccini AR. Novel PGS/PCL electrospun fiber mats with patterned topographical features for cardiac patch applications. Mater Sci Eng C Mater Biol Appl. 2016;69:569–576. 27612749 10.1016/j.msec.2016.06.083

[29] Fang X, Gustafsson AB. DRP1-mediated mitophagy: Safeguarding obese hearts from cardiomyopathy. Circ Res. 2023;133(1):22–24. 37347834 10.1161/CIRCRESAHA.123.323013 PMC10338021

[30] Sidorov VY, Samson PC, Sidorova TN, Davidson JM, Lim CC, Wikswo JP. I-wire heart-on-a-Chip I: Three-dimensional cardiac tissue constructs for physiology and pharmacology. Acta Biomater. 2017;48:68–78. 27818308 10.1016/j.actbio.2016.11.009 PMC5235983

[31] Liu Y, Deng W, Yang L, Fu X, Wang Z, van Rijn P, Zhou Q, Yu T. Biointerface topography mediates the interplay between endothelial cells and monocytes. RSC Adv. 2020;10(23):13848–13854. 35492981 10.1039/d0ra00704h PMC9051607

[32] Yin Z, Chen X, Chen JL, Shen WL, Hieu Nguyen TM, Gao L, Ouyang HW. The regulation of tendon stem cell differentiation by the alignment of nanofibers. Biomaterials. 2010;31(8):2163–2175. 19995669 10.1016/j.biomaterials.2009.11.083

[33] Bae WG, Kim J, Choung YH, Chung Y, Suh KY, Pang C, Chung JH, Jeong HE. Bio-inspired configurable multiscale extracellular matrix-like structures for functional alignment and guided orientation of cells. Biomaterials. 2015;69:158–164. 26285083 10.1016/j.biomaterials.2015.08.006

[34] Zhou QH, Zhao ZY, Zhou ZW, Zhang G, Chiechi RC, van Rijn P. Directing mesenchymal stem cells with gold nanowire arrays. Adv Mater Interfaces. 2018;5(14):1800334.

[35] Shah M, Chacko LA, Joseph JP, Ananthanarayanan V. Mitochondrial dynamics, positioning and function mediated by cytoskeletal interactions. Cell Mol Life Sci. 2021;78(8):3969–3986. 33576841 10.1007/s00018-021-03762-5 PMC11071877

[36] Wolf C, Pouya A, Bitar S, Pfeiffer A, Bueno D, Rojas-Charry L, Arndt S, Gomez-Zepeda D, Tenzer S, Bello FD, et al. GDAP1 loss of function inhibits the mitochondrial pyruvate dehydrogenase complex by altering the actin cytoskeleton. Commun Biol. 2022;5(1):541. 35662277 10.1038/s42003-022-03487-6 PMC9166793

[37] Geng J, Kang Z, Sun Q, Zhang M, Wang P, Li Y, Li J, Su B, Wei Q. Microtubule assists Actomyosin to regulate cell nuclear mechanics and chromatin accessibility. Research. 2023;6:0054. 37040508 10.34133/research.0054 PMC10076026

[38] Hou XS, Wang HS, Mugaka BP, Yang GJ, Ding Y. Mitochondria: Promising organelle targets for cancer diagnosis and treatment. Biomater Sci. 2018;6(11):2786–2797. 30182102 10.1039/c8bm00673c

[39] Zhuang J, Chen L, Li G, Xia L, Wu S, Leng J, Tao X, Hong J, Wu Y, Wang S, et al. RCAN1 deficiency aggravates sepsis-induced cardiac remodeling and dysfunction by accelerating mitochondrial pathological fission. Inflamm Res. 2022;71(12):1589–1602. 36305917 10.1007/s00011-022-01628-5

[40] Yue P, Zhang Y, Liu L, Zhou K, Xia S, Peng M, Yan H, Tang X, Chen Z, Zhang D, et al. Yap1 modulates cardiomyocyte hypertrophy via impaired mitochondrial biogenesis in response to chronic mechanical stress overload. Theranostics. 2022;12(16):7009–7031. 36276651 10.7150/thno.74563 PMC9576622

[41] Ling G, Wang X, Tan N, Cao J, Li W, Zhang Y, Jiang J, Sun Q, Jiang Y, Wang W, et al. Mechanisms and drug intervention for doxorubicin-induced cardiotoxicity based on mitochondrial bioenergetics. Oxidative Med Cell Longev. 2022;2022:7176282. 10.1155/2022/7176282 PMC9586735 36275901

[42] Loson OC, Song Z, Chen H, Chan DC. Fis1, Mff, MiD49, and MiD51 mediate Drp1 recruitment in mitochondrial fission. Mol Biol Cell. 2013;24(5):659–667. 23283981 10.1091/mbc.E12-10-0721 PMC3583668

[43] Altounian G. Bonding of orthodontic brackets. 2: Bonding of brackets using the indirect method. Cah Prothese. 1986;14(56):135–164. 3533231

[44] Cao YL, Meng S, Chen Y, Feng JX, Gu DD, Yu B, Li YJ, Yang JY, Liao S, Chan DC, et al. MFN1 structures reveal nucleotide-triggered dimerization critical for mitochondrial fusion. Nature. 2017;542(7641):372–376. 28114303 10.1038/nature21077 PMC5319402

[45] Gruszczyk AV, Casey AM, James AM, Prag HA, Burger N, Bates GR, Hall AR, Allen FM, Krieg T, Saeb-Parsy K, et al. Mitochondrial metabolism and bioenergetic function in an anoxic isolated adult mouse cardiomyocyte model of in vivo cardiac ischemia-reperfusion injury. Redox Biol. 2022;54: Article 102368. 35749842 10.1016/j.redox.2022.102368 PMC9234472

[46] Chen W, Zhao H, Li Y. Mitochondrial dynamics in health and disease: Mechanisms and potential targets. Signal Transduct Target Ther. 2023;8(1):333. 37669960 10.1038/s41392-023-01547-9 PMC10480456

[47] Giacomello M, Pyakurel A, Glytsou C, Scorrano L. The cell biology of mitochondrial membrane dynamics. Nat Rev Mol Cell Biol. 2020;21(4):204–224. 32071438 10.1038/s41580-020-0210-7

[48] Shaughnessy DT, McAllister K, Worth L, Haugen AC, Meyer JN, Domann FE, van Houten B, Mostoslavsky R, Bultman SJ, Baccarelli AA, et al. Mitochondria, energetics, epigenetics, and cellular responses to stress. Environ Health Perspect. 2014;122(12):1271–1278. 25127496 10.1289/ehp.1408418 PMC4256704

[49] Diebold L, Chandel NS. Mitochondrial ROS regulation of proliferating cells. Free Radic Biol Med. 2016;100:86–93. 27154978 10.1016/j.freeradbiomed.2016.04.198

[50] Franchina DG, Dostert C, Brenner D. Reactive oxygen species: Involvement in T cell signaling and metabolism. Trends Immunol. 2018;39(6):489–502. 29452982 10.1016/j.it.2018.01.005

[51] Ma C, Kuzma ML, Bai X, Yang J. Biomaterial-based metabolic regulation in regenerative engineering. Adv Sci. 2019;6(19):1900819. 10.1002/advs.201900819 PMC6774061 31592416

[52] Zaky HS, el-Said NT, Aboutaleb AS, Allam A, Mansour M, Ahmed HI, Abdel-Sattar SA. Mito-TEMPO mitigates fibromyalgia induced by reserpine in rats: Orchestration between SIRT1, mitochondrial dynamics, endoplasmic reticulum and miRNA-320. Neurochem Res. 2025;50(3):172. 40434586 10.1007/s11064-025-04424-9 PMC12119751

[53] Wang N, Wang X, Lan B, Gao Y, Cai Y. DRP1, fission and apoptosis. Cell Death Discov. 2025;11(1):150. 40195359 10.1038/s41420-025-02458-0 PMC11977278

[54] Chang X, Niu S, Shang M, Li J, Guo M, Zhang W, Sun Z, Li Y, Zhang R, Shen X, et al. ROS-Drp1-mediated mitochondria fission contributes to hippocampal HT22 cell apoptosis induced by silver nanoparticles. Redox Biol. 2023;63: Article 102739. 37187014 10.1016/j.redox.2023.102739 PMC10199224

[55] Lu S, Baier MJ, Polonen RP, Liao Z, Martin JL, Ginsburg KS, Mustroph J, Bers DM. Hyperglycaemia-induced reactive oxygen species production in cardiac ventricular myocytes differs among mammals. J Physiol. 2025; 10.1113/JP287886. 10.1113/JP287886 PMC13189649 40349305

[56] Zheng J, Zhao L, Liu Y, Chen M, Guo X, Wang J. N-acetylcysteine, a small molecule scavenger of reactive oxygen species, alleviates cardiomyocyte damage by regulating OPA1-mediated mitochondrial quality control and apoptosis in response to oxidative stress. J Thorac Dis. 2024;16(8):5323–5336. 39268103 10.21037/jtd-24-927 PMC11388216

[57] Yu T, Li X, Wang C, Yang Y, Fu X, Li T, Wang W, Liu X, Jiang X, Wei D, et al. Lactylation of mitochondrial adenosine triphosphate synthase subunit alpha regulates vascular remodeling and progression of aortic dissection. Research. 2025;8:0799. 40800583 10.34133/research.0799 PMC12342782

[58] Crosas-Molist E, Graziani V, Maiques O, Pandya P, Monger J, Samain R, George SL, Malik S, Salise J, Morales V, et al. AMPK is a mechano-metabolic sensor linking cell adhesion and mitochondrial dynamics to myosin-dependent cell migration. Nat Commun. 2023;14(1):2740. 37217519 10.1038/s41467-023-38292-0 PMC10202939

[59] Yang W, Li X, He L, Zhu S, Lai S, Zhang X, Huang Z, Yu B, Cui C, Wang Q. Empagliflozin improves renal ischemia-reperfusion injury by reducing inflammation and enhancing mitochondrial fusion through AMPK-OPA1 pathway promotion. Cell Mol Biol Lett. 2023;28(1):42. 37202752 10.1186/s11658-023-00457-6 PMC10197452

[60] Carling D. AMPK signalling in health and disease. Curr Opin Cell Biol. 2017;45:31–37. 28232179 10.1016/j.ceb.2017.01.005

[61] Herzig S, Shaw RJ. AMPK: Guardian of metabolism and mitochondrial homeostasis. Nat Rev Mol Cell Biol. 2018;19(2):121–135. 28974774 10.1038/nrm.2017.95 PMC5780224

[62] Wang Y, Li Y, Chen S, Yu T, Sun W, Liu J, Ren H, Zhou Y, Wang L, Tao X, et al. Notch2 signaling drives cardiac hypertrophy by suppressing purine nucleotide metabolism. Research. 2025;8:0635. 40104444 10.34133/research.0635 PMC11913782

[63] Ding Y, Hong T. Low doses of ozone alleviate cardiomyocyte ferroptosis induced by hypoxia–reoxygenation injury via the AMPK–mTOR pathway. Eur J Med Res. 2025;30(1):558. 40604957 10.1186/s40001-025-02829-4 PMC12217297

[64] Tripathy N, Ahmad R, Khang G. Nanomaterial-assisted tissue engineering and regenerative medical therapy. In:Handbook of Intelligent Scaffolds for Tissue Engineering and Regenerative Medicine Jenny Stanford Publishing; 2017. p. 621–656.

[65] Zhou Q, Xie J, Bao M, Yuan H, Ye Z, Lou X, Zhang Y. Engineering aligned electrospun PLLA microfibers with nano-porous surface nanotopography for modulating the responses of vascular smooth muscle cells. J Mater Chem B. 2015;3(21):4439–4450. 32262788 10.1039/c5tb00051c

[66] Wang C, Yu B, Zhou H, Li H, Li S, Li X, Wang W, Feng Y, Yu T. tRF-AspGTC promotes intracranial aneurysm formation by controlling TRIM29-mediated Galectin-3 ubiquitination. Research. 2025;8:0574. 39776588 10.34133/research.0574 PMC11704088

[67] Benard G, Rossignol R. Ultrastructure of the mitochondrion and its bearing on function and bioenergetics. Antioxid Redox Signal. 2008;10(8):1313–1342. 18435594 10.1089/ars.2007.2000

[68] Singh N, NaveenKumar SK, Geethika M, Mugesh G. A cerium vanadate Nanozyme with specific superoxide dismutase activity regulates mitochondrial function and ATP synthesis in neuronal cells. Angew Chem Int Ed Engl. 2021;60(6):3121–3130. 33079465 10.1002/anie.202011711

[69] Agarwal S, Yadav A, Tiwari SK, Seth B, Chauhan LK, Khare P, Ray RS, Chaturvedi RK. Dynamin-related protein 1 inhibition mitigates bisphenol A-mediated alterations in mitochondrial dynamics and neural stem cell proliferation and differentiation. J Biol Chem. 2016;291(31):15923–15939. 27252377 10.1074/jbc.M115.709493 PMC4965546

[70] Jain M, Olsen HE, Paten B, Akeson M. The Oxford Nanopore MinION: Delivery of nanopore sequencing to the genomics community. Genome Biol. 2016;17(1):239. 27887629 10.1186/s13059-016-1103-0 PMC5124260

[71] Krishnakumar R, Sinha A, Bird SW, Jayamohan H, Edwards HS, Schoeniger JS, Patel KD, Branda SS, Bartsch MS. Systematic and stochastic influences on the performance of the MinION nanopore sequencer across a range of nucleotide bias. Sci Rep. 2018;8(1):3159. 29453452 10.1038/s41598-018-21484-w PMC5816649

