# Supplementary material for: Anisotropic Micro/Nanotopography Regulating Mitochondrial Dynamics in Cardiomyocytes
Source: Research (Wash D C). 2025 Sep 16;2025:0891. doi: 10.34133/research.0891 (PMC12439428; doi:10.34133/research.0891)
Supplement: Supplementary 1 — Figs. S1 to S5 [file research.0891.f1.docx]

**Supporting Information**

**Anisotropic Micro/Nanotopography Regulating Mitochondrial Dynamics in** **Cardiomyocytes**

Yan Liu^1,2#^, Bingcheng Yi^3#^, Liangliang Yang^4^, Yanyan Yang^5^, Tianxiang Li^1^, Xiaolu Li^6^, Jae Youl Cho^2^*, Dengshen Zhang^7^*, Qihui Zhou^3^*, and Tao Yu^1^*

^1^ Institute for Translational Medicine, The Affiliated Hospital of Qingdao University, Qingdao 266021, People’s Republic of China;

^2^ Department of Integrative Biotechnology, Sungkyunkwan University, 300 Chuncheon-Dong, Suwon 16419, Republic of Korea;

^3^ Qingdao Key Laboratory of Materials for Tissue Repair and Rehabilitation, Shandong Engineering Research Center for Tissue Rehabilitation Materials and Devices, School of Rehabilitation Sciences and Engineering, University of Health and Rehabilitation Sciences, Qingdao 266113, People’s Republic of China;

^4^ School of Pharmaceutical Sciences, Wenzhou Medical University, Wenzhou 325035, China

^5^ Department of Immunology, School of Basic Medicine, Qingdao University, 266021, People’s Republic of China;

^6^ Department of Cardiac Ultrasound, the Affiliated Hospital of Qingdao University, Qingdao 266000, Shandong, People's Republic of China;

^7^ Department of Cardiovascular Surgery, Affiliated hospital of Zunyi Medical University, Zunyi 563000, Guizhou, People’s Republic of China.

* Corresponding author:

Tao Yu, Professor, Tel: +86-532-82991791; Fax: +86-532-82991791; E-mail: yutao0112@qdu.edu.cn

QiHui Zhou, Professor, Tel: +86-17660670299; Email: [qihuizhou@uor.edu.cn](mailto:qihuizhou@uor.edu.cn).

Dengshen Zhang, Professor, Tel: +86-0851-28608701; Email: xwkzds2021@zmu.edu.cn.

Jae Youl Cho, Professor, Tel: +82-31-290-7868; Fax: +82-31-290-7870; E-mail: [jaecho@skku.edu](mailto:jaecho@skku.edu)

^#^ These authors contributed equally to this paper.


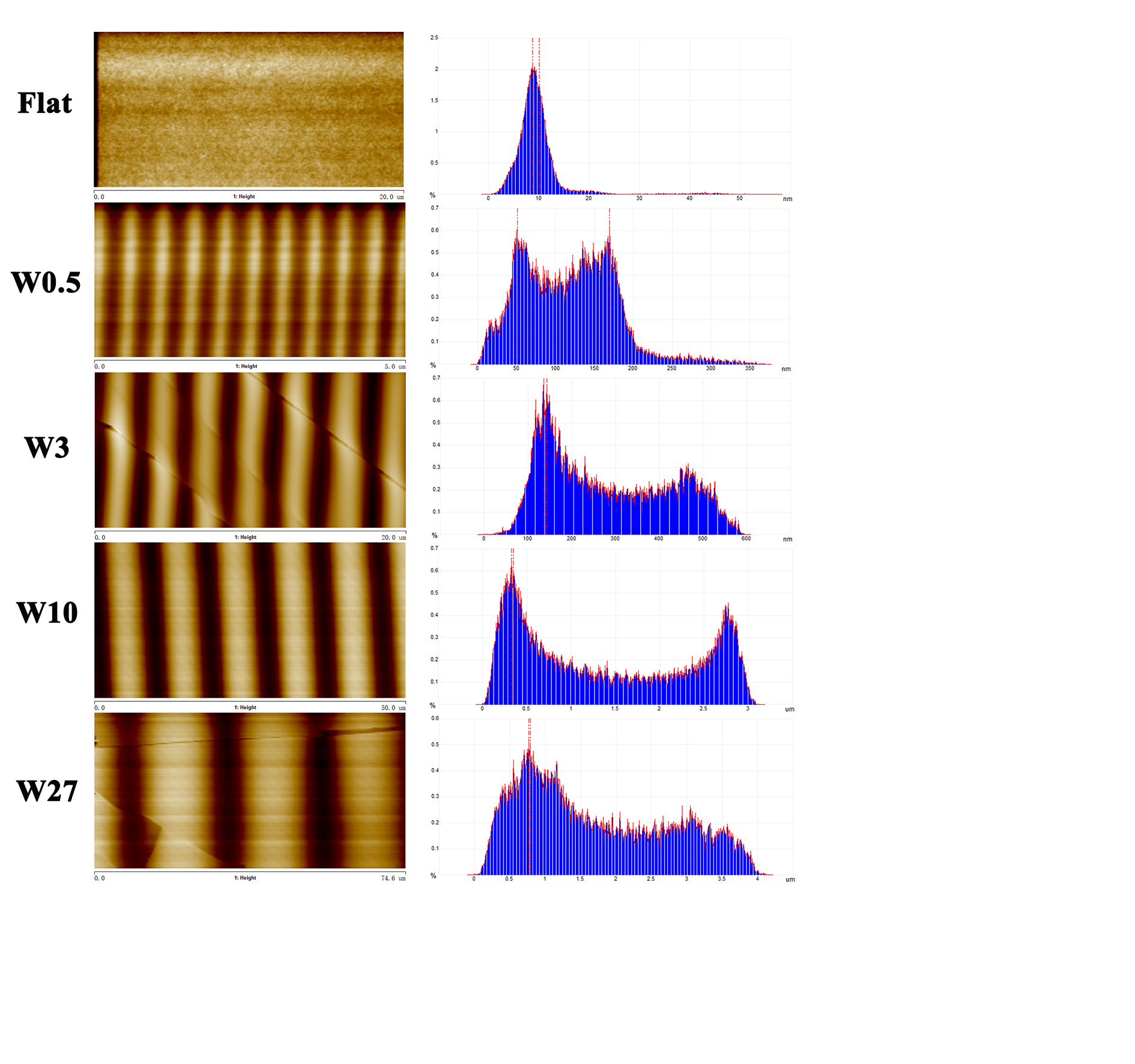


**Supplemental** **Figure 1.** AFM shows the wrinkle size from Flat, W0.5, W3, W10, W27 of PDMS.

**
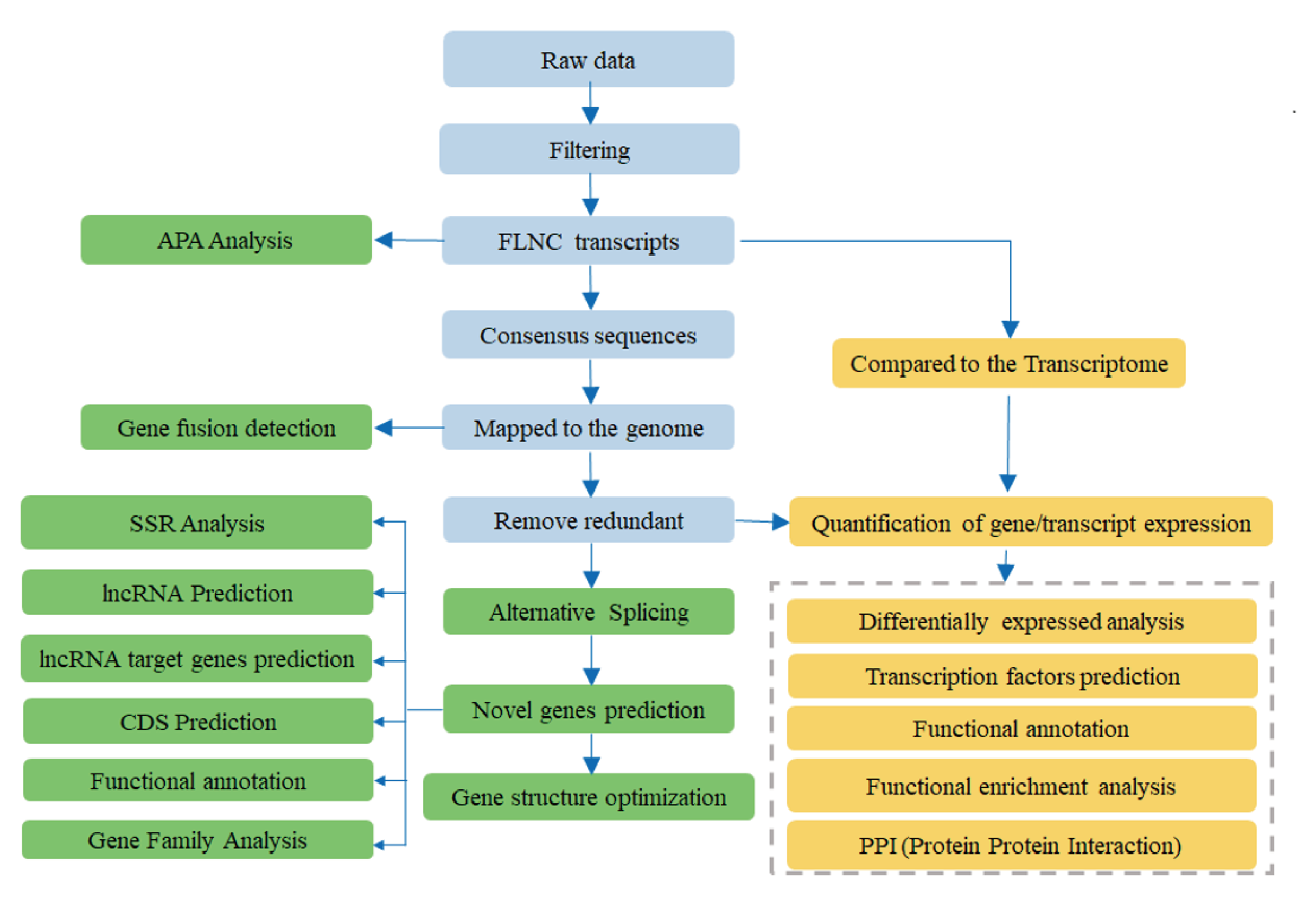
Supplemental** **Figure 2.** Full-length transcriptome bioinformatics workflow.

**Supplemental** **Figure 3. (A)** The volcano plots were performed to provide a quick visualization method of transcripts and genes, displaying the expression alternation between W0.5-treated group and the Flat group. **(B)** The hierarchical cluster indicated the difference of W0.5-treated group and the Flat group. **(C-D)** Bboxplot is used to display CPM (counts per million) distribution. **(E)** Differentially expressed transcripts (DETs) and DEGs. **(F-G)** Statistics of Pathway Enrichment in W0.5 biomaterials.

**
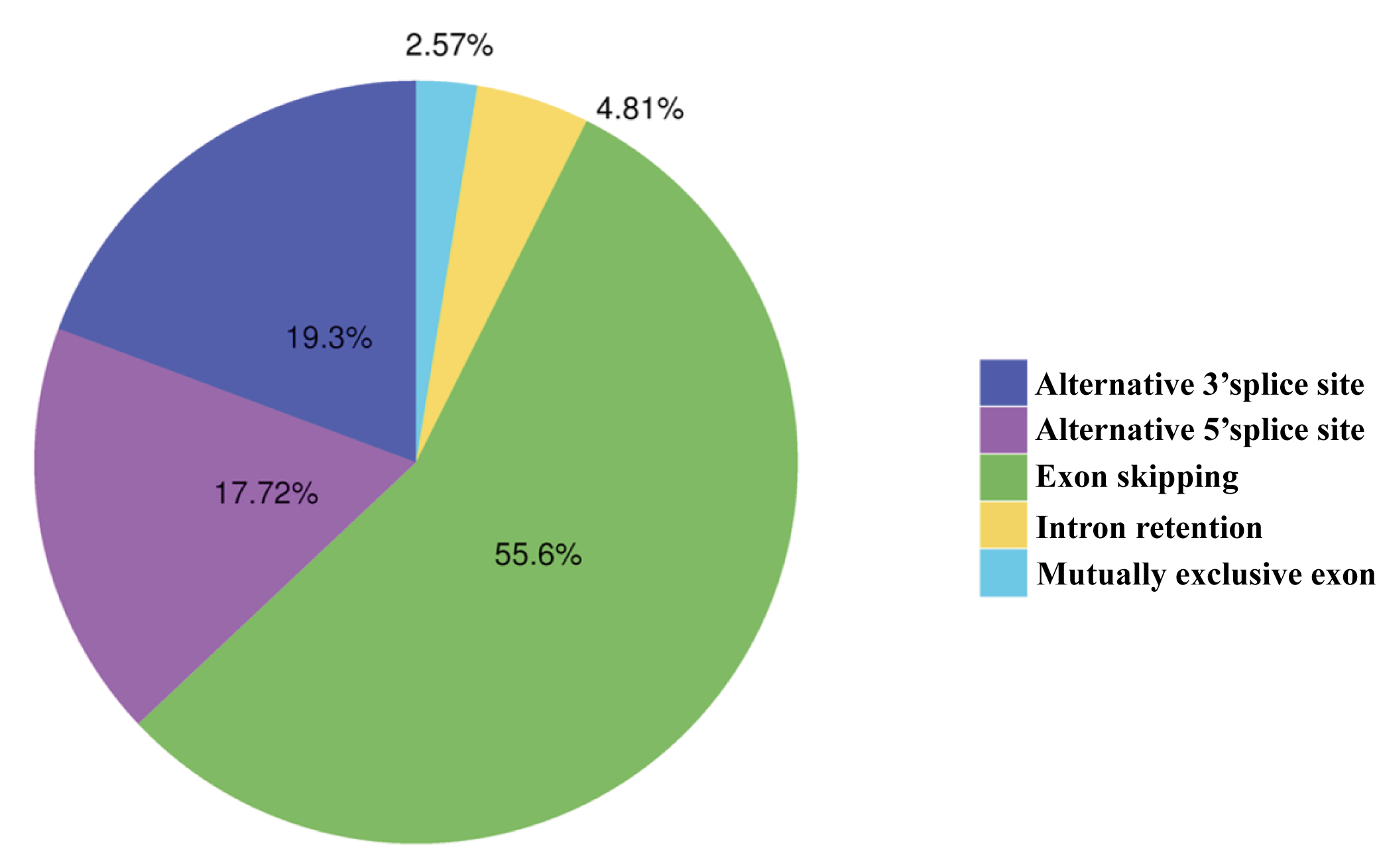
**

**Supplemental** **Figure 4. Statistical diagram of alternative splicing event number.**

**
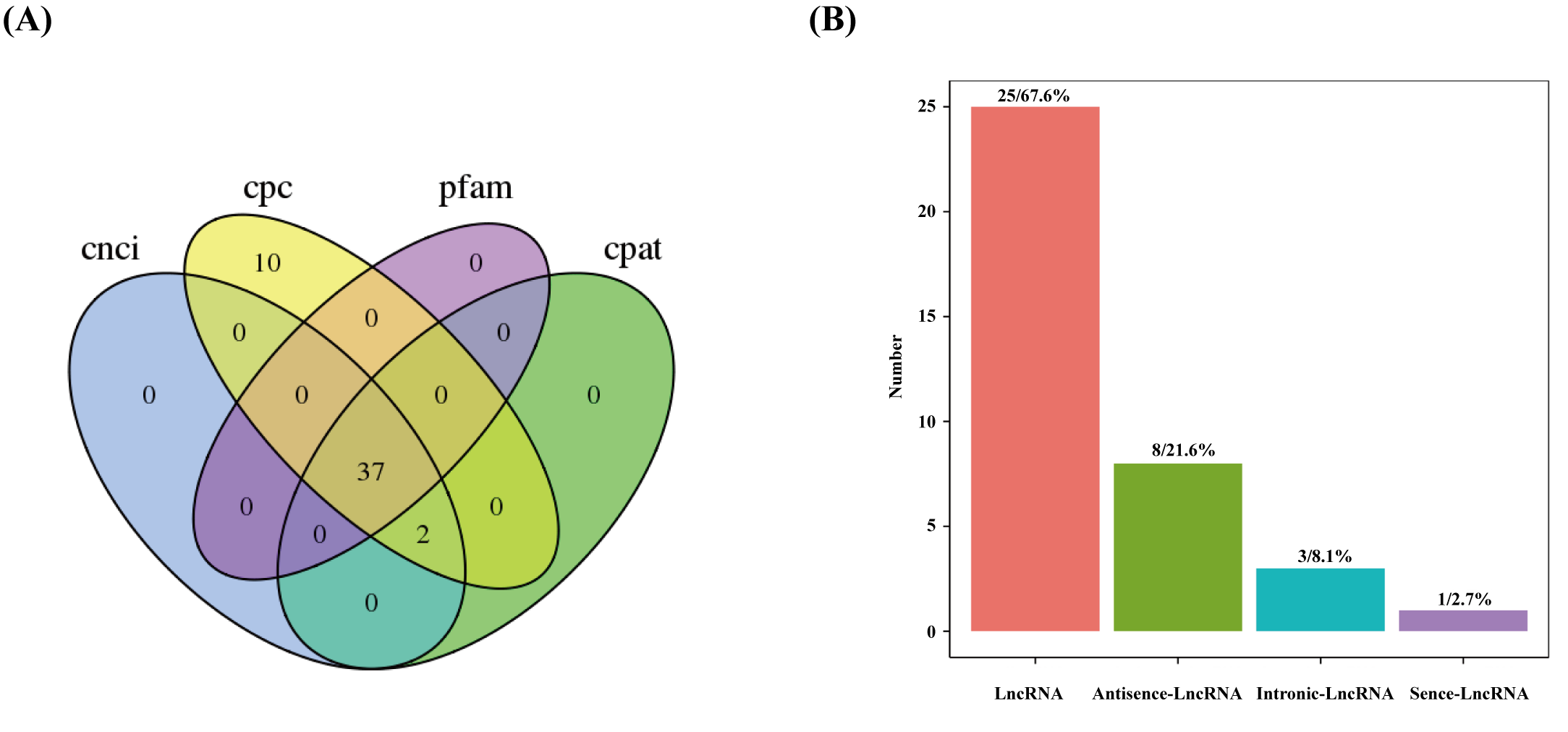
**

**Supplemental** **Figure 5. (A)** LncRNA classification map. **(B)** LncRNA transcripts were predicted by all four methods.
